# Body Mass Index and Risks of More Than 40 Cause‐Specific Mortality in Chinese Women: A Prospective Cohort Study

**DOI:** 10.1002/jcsm.70330

**Published:** 2026-07-16

**Authors:** Jingcen Hu, Yalei Ke, Canqing Yu, Dianjianyi Sun, Yuanjie Pang, Pei Pei, Ling Yang, Yiping Chen, Huaidong Du, Xiaoyu Chang, Maxim Barnard, Junshi Chen, Zhengming Chen, Liming Li, Jun Lv, China Kadoorie Biobank Collaborative Group

**Affiliations:** ^1^ Department of Epidemiology & Biostatistics, School of Public Health Peking University Beijing China; ^2^ Peking University Center for Public Health and Epidemic Preparedness & Response Beijing China; ^3^ Key Laboratory of Epidemiology of Major Diseases (Peking University), Ministry of Education Beijing China; ^4^ Clinical Trial Service Unit and Epidemiological Studies Unit (CTSU), Nuffield Department of Population Health University of Oxford Oxford UK; ^5^ NCDs Prevention and Control Department Sichuan CDC Sichuan China; ^6^ China National Center for Food Safety Risk Assessment Beijing China; ^7^ State Key Laboratory of Vascular Homeostasis and Remodeling Peking University Beijing China

**Keywords:** body mass index, obesity, premature death, underweight, women

## Abstract

**Background:**

The health consequences of being underweight are less well‐studied than obesity. We aimed to examine the association between body mass index (BMI) and mortality from a wide range of diseases across the full BMI spectrum, with particular focus on how low BMI may affect premature and nonpremature death.

**Methods:**

After excluding ever‐smokers and those with pre‐existing major diseases, this study included 262 704 women aged 30–79 from the China Kadoorie Biobank. The baseline BMI (kg/m^2^) was calculated using measured height and weight and classified into six groups: < 18.5, 18.5–19.9, 20.0–22.4, 22.5–23.9 (reference), 24.0–27.9 and ≥ 28.0. Long‐term follow‐up was conducted by linking to the local death registry. Cox regression was used to estimate the hazard ratios (HRs) of BMI with all‐cause and cause‐specific mortality, after adjusting for waist circumference and potential confounders.

**Results:**

At baseline, 3.9%, 7.8% and 11.4% of participants had BMIs (kg/m^2^) of < 18.5, 18.5–19.9 and ≥ 28.0, respectively. During a median follow‐up of 17.1 years, 29 531 deaths were recorded, including 11 455 premature deaths (under the age of 70) and 18 076 nonpremature deaths. The risk of all‐cause mortality significantly increased at the lower extreme of BMI, reached a nadir around 23.5 kg/m^2^ estimated from restricted cubic splines and showed a modest increase at the upper extreme. Participants with BMI < 18.5, 18.5–19.9 and 20.0–22.4 had higher risks of premature death (HRs) of 1.91 (1.73–2.10), 1.24 (1.14–1.34) and 1.16 (1.11–1.21), respectively. The corresponding HRs for nonpremature death were 1.46 (1.36–1.56), 1.17 (1.10–1.23) and 1.04 (1.01–1.08). The analysis of premature death involved 33 diseases from eight ICD‐10 chapters, and nonpremature death involved 40 diseases from nine chapters. Underweight was linked to an increased risk of premature death in seven chapters and 10 diseases, including neoplasms (HR = 1.36, 95% CI: 1.16–1.59), endocrine‐metabolic (6.03, 4.13–8.82), circulatory (1.85, 1.56–2.20), respiratory (5.85, 3.89–8.80), digestive (5.64, 3.13–10.18), genitourinary (2.61, 1.20–5.69) and external causes (2.06, 1.55–2.72). Underweight was also associated with an increased risk of nonpremature death in four chapters and seven diseases. In contrast, obesity was only associated with increased risks of premature and nonpremature death in two and one chapters, respectively.

**Conclusion:**

Among Chinese female healthy never‐smokers, underweight was an important risk factor for all‐cause and multiple cause‐specific mortality, especially the risk of premature death. While addressing the global obesity epidemic, the negative health consequences of low weight should not be ignored.

## 1 Introduction

Underweight and obesity are two extreme forms of malnutrition that pose major challenges to global public health [1]. In 2022, approximately 347 million adults worldwide were underweight, and 878 million were obese, with women accounting for 183 million and 504 million, respectively. The body mass index (BMI) distribution varied across countries with different levels of economic development [2]. In 2017, the average BMI (kg/m^2^) for urban and rural women in East and Southeast Asia was 23.3 and 23.9, respectively, whereas in high‐income Western countries, urban and rural women had an average BMI of 26.9 [3]. In 2022, the global standardized prevalence of underweight was 7.2%, with rates of 14.3% in low‐income countries and 3.4% in high‐income countries [4].

Numerous cohort studies have linked overweight and obesity, as well as underweight, to an increased mortality risk [5, 6]. However, most studies were conducted in developed countries [5, 6], providing more solid evidence on the association between high BMI and mortality risk. In contrast, the findings on the relationship between low BMI and mortality risk remain inconsistent. Furthermore, previous studies on the association between BMI and cause‐specific mortality, which were limited by sample size, typically grouped causes of death broadly [6, 7, 8, 9, 10]. Only a few studies have used a consistent analytical strategy, including BMI classification criteria, model specifications and covariate adjustment, within the same population to investigate the relationship between BMI and multiple specific causes of death across all organ systems [11, 12, 13], with only one study reporting results for underweight and low‐normal weight [13]. It is also worth mentioning that smoking and prevalent or undiagnosed illnesses may bias the analysis of BMI and mortality. Conventional covariate adjustment, which has been used in many previous studies investigating the association between BMI and mortality risk, was considered challenging to control for these biases fully [5]. Stricter strategies, such as excluding ever‐smokers, participants with prevalent disease and early follow‐up, are recommended, especially for low BMI analysis.

Underweight remains a significant public health concern in developing countries or regions. Even in developed countries, cases of underweight exist due to intentional weight loss [14]. However, there are significant evidence gaps regarding the health consequences of the low end of the BMI spectrum. Using data from the China Kadoorie Biobank (CKB) of 0.3 million women over a 20‐year follow‐up, this study aimed to examine the association between BMI and mortality from a wide range of diseases across the full BMI spectrum, with particular focus on the potentially different impacts of low BMI on the risks of premature death and death at or after the age of 70. The large sample size and adequate mortality allowed us to perform the analysis among healthy never‐smokers. We also adjusted for waist circumference (WC) to ensure that the association was independent of central adiposity, thereby better reflecting the role of lean body mass.

## 2 Methods

### 2.1 Study Population

The study design and implementation for CKB have been previously reported [15, 16]. Briefly, 210 205 men and 302 519 women aged 30–79 were recruited from 10 geographically diverse (five urban and five rural) areas in China between 2004 and 2008. Trained staff used laptop‐based questionnaires to gather baseline information, undertook physical measurements and collected blood samples. Following the baseline survey, periodic resurveys were conducted every 4–5 years, with around 5% of the surviving participants randomly chosen from the original cohort, and the same data were collected as at baseline. All participants were followed up immediately after recruitment using electronic linkages to established disease and death registries and national health insurance databases. By 31 December 2023, only 3843 participants (0.7%) were lost to follow‐up.

The Ethical Review Committee of the Chinese Centre for Disease Control and Prevention (Beijing, China), the Peking University Health Science Center (Beijing, China) and the Oxford Tropical Research Ethics Committee, University of Oxford (UK), approved the study. All participants provided written informed consent.

### 2.2 Assessment of BMI

At baseline, trained staff took anthropometric measurements according to a standard protocol. Height was measured using a stadiometer, and weight was recorded with a TANITA‐TBF‐300GS scale, with participants wearing light clothing and without shoes, typically to the nearest 0.1 cm or 0.1 kg. The BMI was calculated by dividing weight in kilogrammes by the square of height in metres. The Spearman correlation coefficients for height, weight and BMI among the 19 802 participants in the 2008 resurvey and the baseline survey were 0.99, 0.96 and 0.93, respectively [16]. Based on the BMI cut‐off values set in the Chinese Criteria of Weight for Adults [17] and previous references, BMI (kg/m^2^) was categorized into six groups: < 18.5 (underweight), 18.5–19.9, 20.0–22.4, 22.5–23.9 (the reference group), 24.0–27.9 (overweight) and ≥ 28.0 (obesity).

### 2.3 Assessment of Other Covariates

Questionnaires were used to gather baseline sociodemographic characteristics (age, sex, region, marital status, education, household income and occupation), lifestyle factors (smoking status, alcohol consumption, intakes of fresh fruits, vegetables, and red meat and physical activity) and medical history. WC was measured with nonstretchable tape at the midpoint between the lowest rib and the iliac crest. The forced expiratory volume in 1 s (FEV1) and forced vital capacity (FVC) of the participants were assessed in a sitting position using a handheld electronic spirometer to the nearest 10 mL, without the administration of bronchodilators.

### 2.4 Ascertainment of Outcomes

During the long‐term follow‐up, death information for CKB participants was obtained from the Chinese Disease Surveillance Points system. The causes of death were derived mainly from official death certificates, supplemented by reviewing medical records or conducting standardized verbal autopsy procedures when necessary. All underlying causes of death were coded using the International Classification of Diseases, 10th revision (ICD‐10), by trained medical professionals.

To conduct a ‘phenome‐wide’ analysis, we reviewed all documented deaths coded with three‐character ICD‐10 codes, which were combined, where appropriate, based on disease characteristics. Two ICD‐10 chapters that were considered irrelevant to the study population were excluded: perinatal‐origin diseases (Chapter XVI) and congenital conditions (Chapter XVII). Referring to previous PheWAS studies conducted in CKB [18, 19], the number of deaths in the ICD‐10 chapters or specific outcomes (or small number of specific disease groups) analysed independently needed to be at least 80 to ensure reasonable statistical power. Causes with fewer than 80 deaths were grouped into a ‘less‐common’ category within each ICD‐10 chapter to allow for exploratory analyses. It is worth mentioning that in the primary analysis, we used E11 and E14 to define type 2 diabetes. After excluding deaths clearly not caused by type 2 diabetes and given that the majority of the participants were over the age of 40, deaths coded by E14 were more likely to be type 2 diabetes‐related.

The study outcomes included three categories: all‐cause mortality, chapter‐specific mortality (e.g., I00‐I99, diseases of the circulatory system) and cause‐specific mortality (e.g., I21‐I23, myocardial infarction). The ICD‐10 codes for each outcome are detailed in Table S1. Furthermore, the deaths were also distinguished in the analysis between premature death (death before the age of 70) and death at or after the age of 70.

### 2.5 Statistical Analysis

The participant selection flow chart is presented in Figure S1. We excluded participants with missing or extreme BMI values, self‐reported histories of cardiovascular diseases or cancer and those with self‐reported or screen‐detected chronic obstructive pulmonary disease (COPD) at baseline, primarily to minimize reverse causation. Furthermore, to eliminate the confounding effect of smoking, all ever‐smokers were excluded. This exclusion strategy resulted in a substantial gender imbalance, attributed to the markedly higher smoking prevalence among Chinese men: 47 342 male never‐smokers remained compared with 262 704 female never‐smokers. Consequently, given that the male never‐smoker sample was substantially underpowered for the disease‐specific mortality analyses, all subsequent analyses were restricted to female never‐smokers.

The baseline characteristics of participants across different BMI groups were reported as means and percentages, adjusted for age and region, except for these two variables, using multiple linear regression for continuous variables and logistic regression for binary variables. Linear trend tests were performed using the median value of each BMI group.

Participants in this study were censored on death, loss to follow‐up or 31 December 2023, whichever occurred first. Cox proportional hazard models were used to estimate the HRs for mortality risk associated with different BMI groups. The models were stratified by age‐at‐risk (5‐year groups between 30 and 100 years) and 10 areas and adjusted for marital status, education, annual household income, occupation, alcohol consumption, physical activity, dietary habits (frequency of fruit, vegetable and red meat intake) and WC. We tested the proportional hazards assumption using Schoenfeld residuals and found no significant violation. To facilitate comparisons in analyses with more than two exposure groups, the variance of the log risk for each group, including the reference group, was calculated to obtain group‐specific 95% CIs [20]. We also used restricted cubic splines fitted to Cox models to examine the non‐linear relationship between BMI and the risk of all‐cause mortality.

Several sensitivity analyses were performed: (1) excluding participants with pre‐existing diseases at baseline (diabetes, tuberculosis, hepatitis/cirrhosis, peptic ulcer, gallstones/gallbladder disease and chronic kidney disease) for all‐cause mortality analyses; (2) excluding participants with the aforementioned relevant pre‐existing diseases at baseline for the respective outcomes for chapter‐ and cause‐specific mortality analyses; and (3) excluding participants who died within the first 5 years of follow‐up.

The statistical significance level was set at two‐tailed *p* < 0.05. The all‐cause and chapter‐specific mortality analyses used conventional *p*‐values. FDR‐adjusted *p*‐values [21] were used in the cause‐specific mortality analyses to control the increase in false positive rate caused by multiple testing [22, 23]. All analyses were conducted using the R software version 3.6.0.

## 3 Results

### 3.1 Participants

Among the 262 704 women, the mean baseline age was 50.4 (±10.1) years, and 56.1% resided in rural areas. The proportion of participants who were underweight and had a BMI of 18.5–19.9 kg/m^2^ was 3.9% and 7.8%, respectively, whereas the proportion of obese participants was 11.4% (Table 1). Compared to participants with a BMI of 22.5–23.9 kg/m^2^, both underweight and obese participants had a higher mean baseline age and lower educational level. As BMI decreased, the proportion of rural residents increased, WC decreased and the prevalence of hypertension and diabetes declined.

**TABLE 1:** Baseline characteristics of female participants according to body mass index (*n* = 262 704).

Characteristic	< 18.5 kg/m^2^	18.5–19.9 kg/m^2^	20.0–22.4 kg/m^2^	22.5–23.9 kg/m^2^	24.0–27.9 kg/m^2^	≥ 28.0 kg/m^2^
Number of participants, *n* (%)	10 304 (3.9)	20 470 (7.8)	66 656 (25.4)	47 290 (18.0)	88 080 (33.5)	29 904 (11.4)
Age at baseline, years	52.4	49.7	49.2	49.8	51.0	52.2
Rural area, %	65.3	63.5	59.7	56.1	53.0	48.7
Married, %	88.5	88.8	89.5	90.2	91.0	90.9
High school and above, %	17.4	19.4	20.1	19.6	18.0	14.9
Household income ≥ 10 000 RMB/year, %	65.9	69.0	70.8	72.1	72.6	72.1
Farmer or factory worker, %	57.9	57.0	54.8	53.1	51.3	48.0
Consuming ≥ 4 days per week of, %						
Fresh fruits	26.0	29.6	31.4	32.4	32.4	32.0
Fresh vegetables	97.8	98.2	98.2	98.3	98.4	98.3
Red meat	41.2	42.9	44.1	44.7	45.4	45.3
Excessive alcohol drinking^a^, %	0.6	0.7	0.7	0.8	1.0	1.0
Total physical activity level, MET h/d	21.1	21.4	21.4	21.2	21.0	20.0
Body mass index, kg/m^2^	17.5	19.3	21.3	23.2	25.6	30.1
Waist circumference, cm	63.9	68.3	73.1	77.7	83.3	92.7
Prevalent hypertension, %	15.2	18.7	23.7	29.1	37.3	50.3
Prevalent diabetes, %	2.6	3.3	4.0	5.0	6.4	8.2

*Note:* All means and percentages were adjusted for age and region, except for these two variables, using multiple linear regression for continuous variables or logistic regression for binary variables. Linear trend tests were performed with the median value within each body mass index group, with all tests yielding *p* < 0.001.

Abbreviation: MET h/d, metabolic equivalent of task hours per day.

^a^ Drinking ≥ 30 g of pure alcohol per day, or previously drinking at least once per week.

During 4 339 953 person‐years of follow‐up (median, 17.1 years; maximum, 19.5 years), 29 531 deaths were recorded, including 11 455 deaths before the age of 70 and 18 076 deaths at or after the age of 70. The analysis of premature death involved 33 diseases from eight ICD‐10 chapters, and the analysis of death at or after the age of 70 involved 40 diseases from nine ICD‐10 chapters.

### 3.2 Association Between BMI and All‐Cause Mortality

**FIGURE 1:**
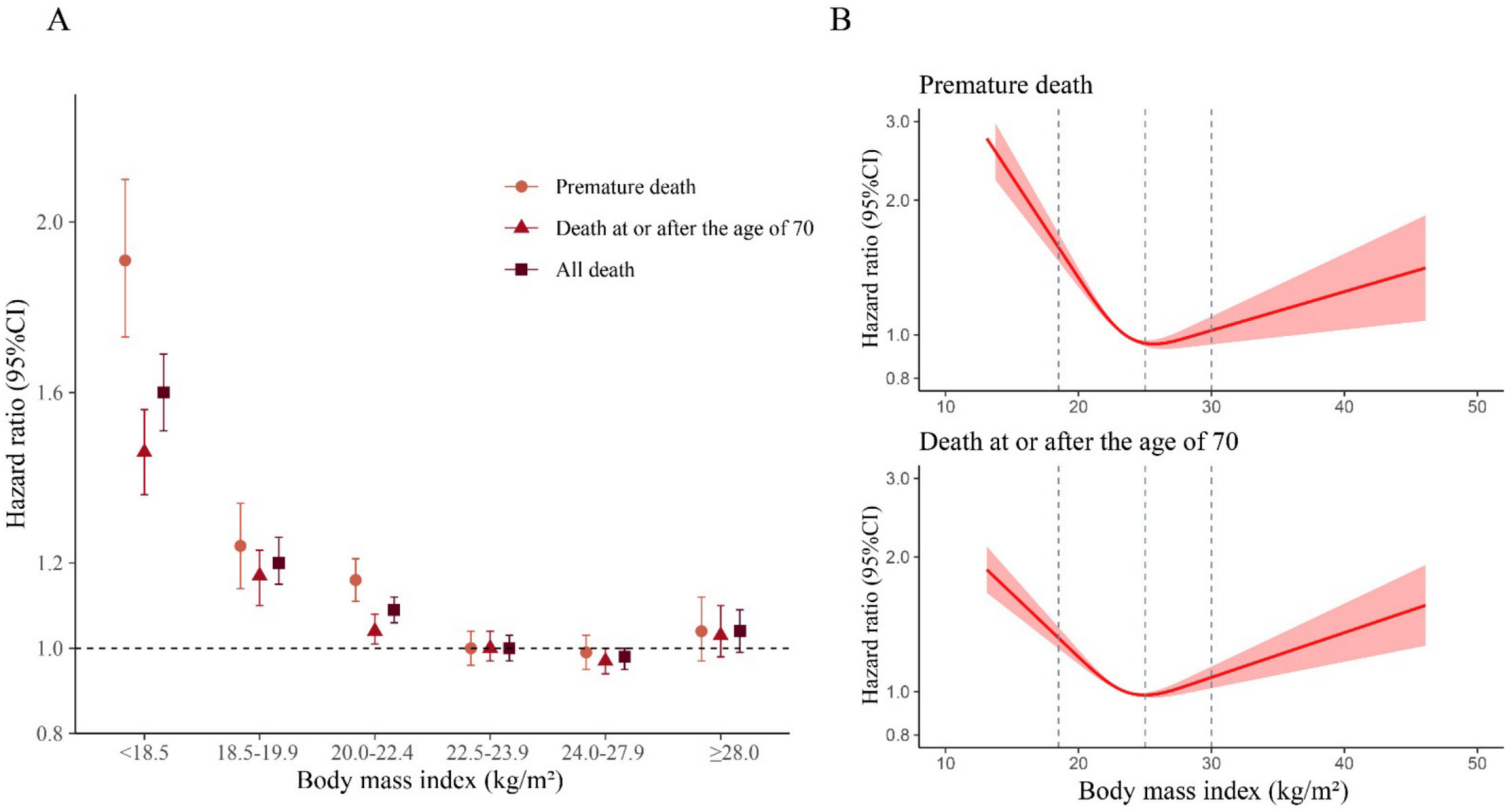
Associations between body mass index and all‐cause mortality. CI, confidence interval. Estimates were adjusted for marital status (married, widowed, separated/divorced and never married), education (no formal school, primary school, middle school, high school, college, university or above), household income (< 2500, 2500–4999, 5000–9999, 10 000–19 999, 20 000–34 999 and ≥ 35 000 yuan), occupation (agriculture and related workers, factory workers, administrator/manager, professional/technical, sales and service workers, retired, housewife/husband, self‐employed, unemployed, other or not stated), alcohol consumption (never, former, weekly but not daily and daily: < 15, 15~, 30~ and ≥ 60 g/d of pure alcohol), physical activity (metabolic equivalent of task [MET] hours per day), dietary habits (frequency of fruit, vegetable and red meat intake; the midpoint value within each group) and waist circumference (cm). Dashed vertical lines in Panel B represent the WHO BMI category thresholds of 18.5 kg/m^2^ (underweight to healthy), 25 kg/m^2^ (healthy weight to overweight) and 30 kg/m^2^ (overweight to obese). The *p*‐values for non‐linearity were less than 0.0001 for both premature death and death at or after the age of 70.

After adjusting for WC and other potential confounding factors such as demographics and lifestyle factors, participants with BMI < 18.5, 18.5–19.9 and 20.0–22.4 kg/m^2^ had a higher all‐cause mortality risk compared to the reference group (22.5–23.9 kg/m^2^) (Figure 1 and Table S2). The HRs for premature death were higher than those for death at or after the age of 70. The HRs for the above three groups on premature death were 1.91 (1.73–2.10), 1.24 (1.14–1.34) and 1.16 (1.11–1.21), respectively, whereas the corresponding HRs for death at or after the age of 70 were 1.46 (1.36–1.56), 1.17 (1.10–1.23) and 1.04 (1.01–1.08). The risk of all‐cause mortality significantly increased at the lower extreme of BMI, reached a nadir around 23.5 kg/m^2^ estimated from restricted cubic splines and showed a modest increase at the upper extreme (Figure 1). When BMI < 23.5 kg/m^2^, the HRs per 5‐kg/m^2^ BMI increase for premature death and death at or after the age of 70 were 0.61 (0.55–0.67) and 0.73 (0.67–0.79), respectively. When BMI ≥ 23.5 kg/m^2^, the corresponding HRs were 1.06 (0.98–1.14) and 1.07 (1.01–1.13), respectively. After excluding those with relevant pre‐existing diseases at baseline or those who died within the first 5 years of follow‐up, the HRs for premature death in the three BMI groups below the reference group were slightly attenuated (Table S2).

### 3.3 Association Between BMI and Chapter‐Specific Mortality

**FIGURE 2:**
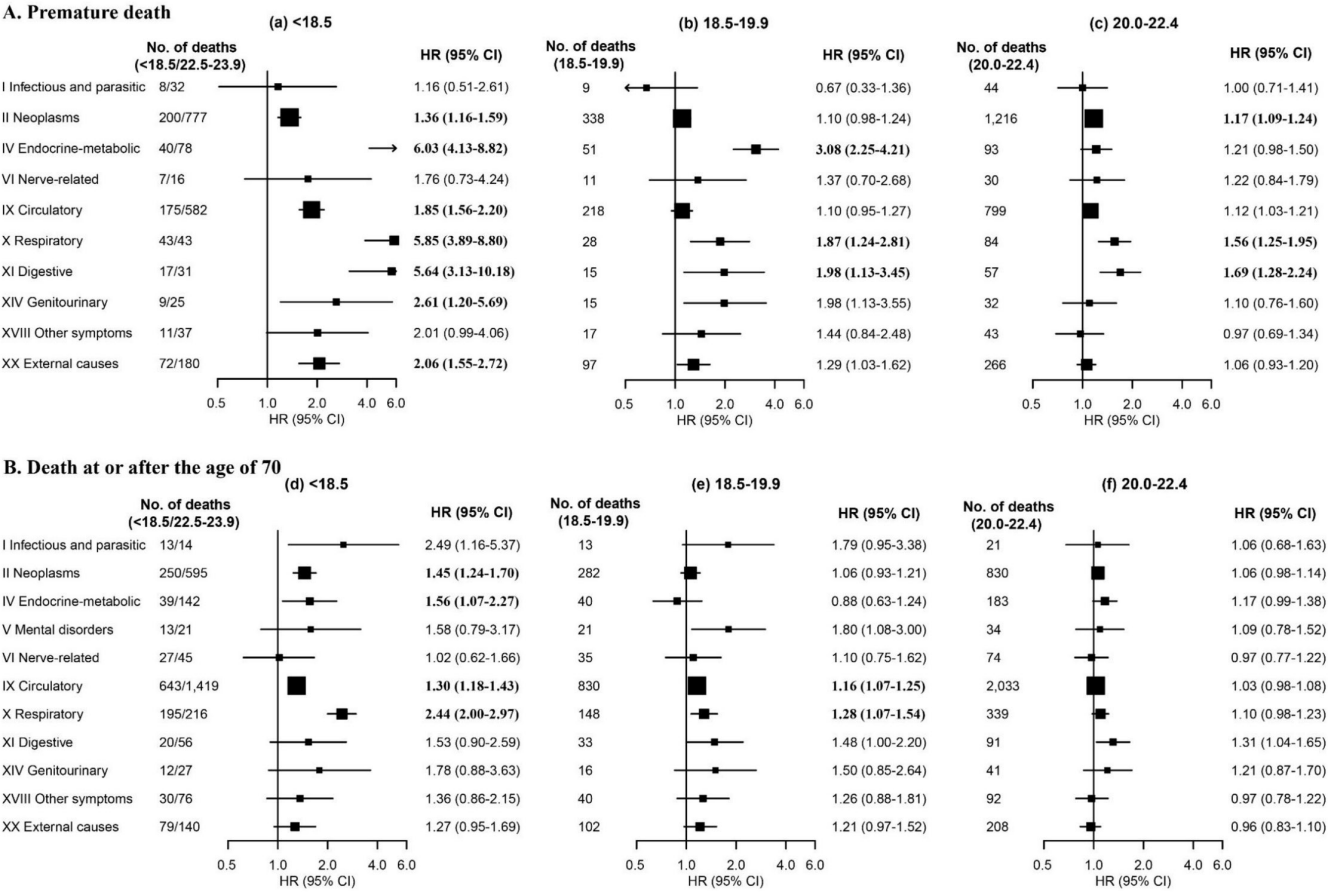
Adjusted HRs for ICD‐10 chapter‐specific mortality associated with body mass index. CI, confidence interval; HR, hazard ratio; ICD‐10, International Classification of Diseases, 10th Revision. The group with a BMI of 22.5–23.9 kg/m^2^ was the reference group. The models were adjusted for the same covariates as described in Figure 1. Each solid square represents HR, with the area inversely proportional to the variance of the log HR. The horizontal lines indicate 95% CIs. Bold indicates *p* < 0.05.

Being underweight was associated with an increased risk of premature death in seven chapters: neoplasms (HR = 1.36, 95% CI: 1.16–1.59), endocrine‐metabolic (6.03, 4.13–8.82), circulatory (1.85, 1.56–2.20), respiratory (5.85, 3.89–8.80), digestive (5.64, 3.13–10.18), genitourinary (2.61, 1.20–5.69) and external causes (2.06, 1.55–2.72) (Figure 2 and Table S3). The other two BMI groups below the reference group had an increased risk of premature death from respiratory and digestive system diseases, but their HRs were slightly lower. Furthermore, a BMI of 18.5–19.9 kg/m^2^ was linked to an increased risk of death from endocrine‐metabolic diseases, and a BMI of 20.0–22.4 kg/m^2^ from neoplasms. We also observed that the obese group had a higher risk of premature death from neoplasms and circulatory system diseases (Table S3).

Underweight was associated with an increased risk of death at or after the age of 70 in four chapters, with the HR for neoplasms slightly exceeding that for premature death; however, the HRs for premature death in the endocrine‐metabolic, circulatory and respiratory chapters were significantly higher than those for death at or after the age of 70 (Figure 2 and Table S3). A BMI of 18.5–19.9 kg/m^2^ was linked to an increased risk of dying at or after the age of 70 from circulatory and respiratory system diseases. Those with obesity were more likely to die at or after the age of 70 from circulatory system diseases (Table S3). The findings of sensitivity analyses are reported in the Supporting Information.

### 3.4 Association Between BMI and Cause‐Specific Mortality

**FIGURE 3:**
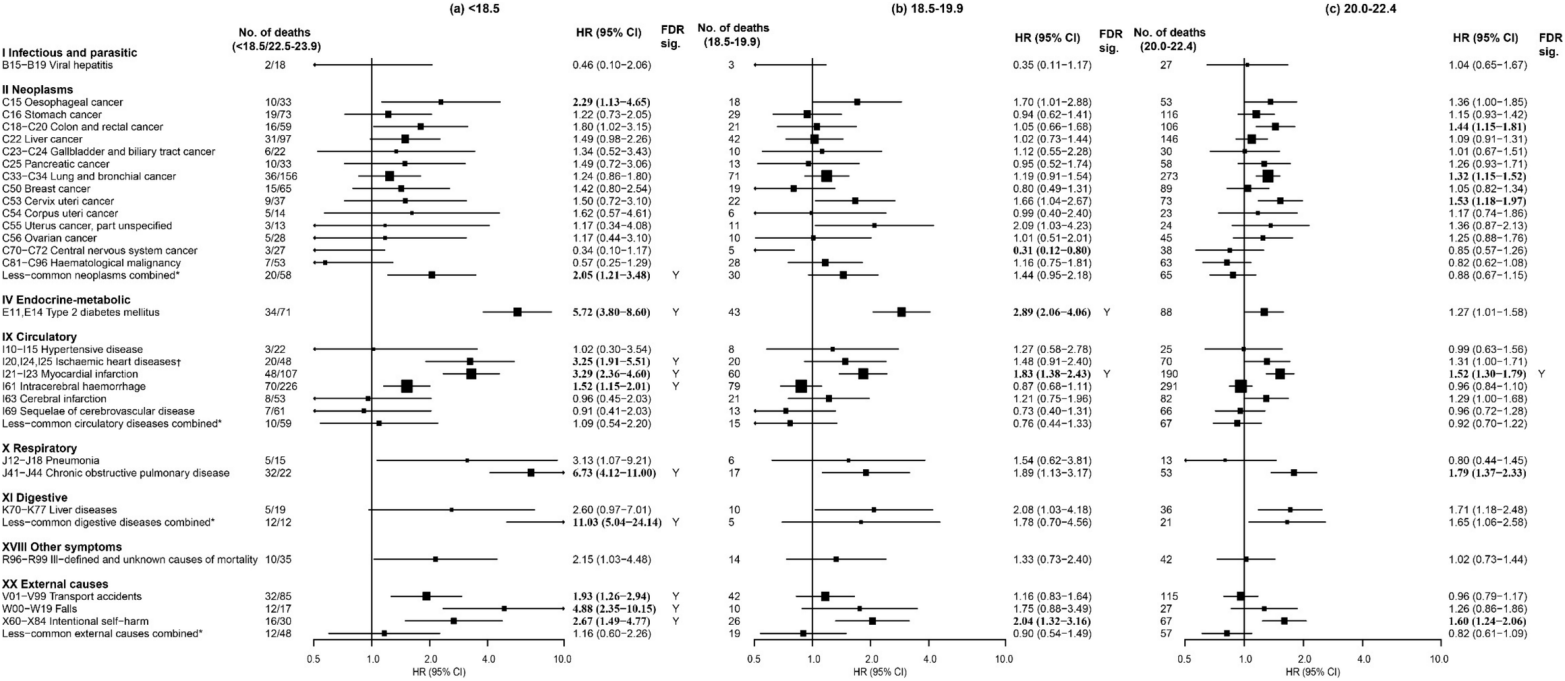
Adjusted HRs for cause‐specific premature death associated with body mass index. CI, confidence interval; HR, hazard ratio; ICD‐10, International Classification of Diseases, 10th Revision. The group with a BMI of 22.5–23.9 kg/m^2^ was the reference group. The models were adjusted for the same covariates as described in Figure 1. Each solid square represents HR, with the area inversely proportional to the variance of the log HR. The horizontal lines indicate 95% CIs. Bold indicates *p* < 0.05. FDR‐adjusted *p* < 0.05 is denoted by ‘Y’ in the ‘FDR sig.’ column. All *p* values are two‐sided. *Included less‐common ICD‐10 codes within the corresponding ICD‐10 chapter that were not individually investigated in the present study. ^†^Ischaemic heart diseases other than myocardial infarction.

There was a statistically significant association between being underweight and an increased risk of premature death for 11 diseases; the association persisted for 10 diseases after FDR adjustment (Table 2). BMI levels of 18.5–19.9 and 20.0–22.4 kg/m^2^ were associated with an increased risk of premature death for two and one disease, respectively. Specifically, compared to participants with a BMI of 22.5–23.9 kg/m^2^, those with BMI < 18.5 and 18.5–19.9 kg/m^2^ were at a higher risk of premature death from type 2 diabetes, with HR (95% CI) of 5.72 (3.80–8.60) and 2.89 (2.06–4.06), respectively (Figure 3 and Table S6). Participants with BMI < 18.5, 18.5–19.9 and 20.0–22.4 kg/m^2^ had an increased risk of premature death from myocardial infarction, with HR (95% CI) of 3.29 (2.36–4.60), 1.83 (1.38–2.43) and 1.52 (1.30–1.79), respectively. Being underweight was positively associated with the risk of premature death from less‐common digestive diseases combined and COPD, with HR (95% CI) of 11.03 (5.04–24.14) and 6.73 (4.12–11.00), respectively. Furthermore, being underweight was also linked to an increased risk of premature death from less‐common neoplasms combined, other ischemic heart disease, intracerebral haemorrhage, transport accidents, falls and intentional self‐harm, with HR (95% CI) ranging from 1.52 (1.15–2.01) to 4.88 (2.35–10.15).

**TABLE 2:** Summary of the number of diseases associated with body mass index by ICD‐10 chapter.

	No. of diseases	< 18.5 kg/m^2^		18.5–19.9 kg/m^2^		20.0–22.4 kg/m^2^		24.0–27.9 kg/m^2^		≥ 28.0 kg/m^2^	
		Positive	Negative	Positive	Negative	Positive	Negative	Positive	Negative	Positive	Negative
Premature death											
I Infectious and parasitic	1	0	0	0	0	0	0	0	0	0	0
II Neoplasms	15	2 (1)	0	0	1 (0)	3 (0)	0	0	0	0	0
IV Endocrine, nutritional and metabolic	1	1 (1)	0	1 (1)	0	0	0	0	1 (0)	0	1 (1)
IX Circulatory	7	3 (3)	0	1 (1)	0	1 (1)	0	0	0	1 (0)	0
X Respiratory	2	1 (1)	0	0	0	1 (0)	0	0	0	0	0
XI Digestive	2	1 (1)	0	0	0	0	0	0	0	0	0
XVIII Other symptoms, signs and abnormal findings	1	0	0	0	0	0	0	0	0	0	0
XX External causes	4	3 (3)	0	1 (0)	0	1 (0)	0	0	0	0	0
Total	33	11 (10)	0	3 (2)	1 (0)	6 (1)	0	0	1 (0)	1 (0)	1 (1)
Death at or after the age of 70											
II Neoplasms	13	3 (2)	1 (0)	1 (0)	1 (0)	0	0	0	0	0	0
IV Endocrine, nutritional and metabolic	2	1 (0)	0	0	0	0	0	0	0	0	1 (0)
VI Nerve‐related	2	0	0	0	0	0	0	0	0	0	0
IX Circulatory	8	3 (2)	0	1 (0)	0	0	0	1 (0)	1 (0)	1 (0)	0
X Respiratory	4	2 (2)	0	0	0	0	0	0	1 (0)	0	0
XI Digestive	2	0	0	1 (0)	0	0	0	0	0	0	0
XIV Genitourinary	2	0	0	0	0	0	0	0	0	0	0
XVIII Other symptoms, signs and abnormal findings	2	0	0	0	0	0	0	0	1 (0)	0	0
XX External causes	5	1 (1)	0	1 (0)	0	0	0	0	0	0	0
Total	40	10 (7)	1 (0)	4 (0)	1 (0)	0	0	1 (0)	3 (0)	1 (0)	1 (0)
All death											
I Infectious and parasitic	2	1 (1)	0	0	0	0	0	0	0	0	0
II Neoplasms	16	4 (3)	1 (1)	2 (0)	0	2 (0)	0	0	0	1 (0)	0
IV Endocrine, nutritional and metabolic	2	2 (1)	0	2 (0)	0	1 (0)	0	0	1 (1)	0	1 (1)
VI Nerve‐related	2	0	0	0	0	0	0	0	0	0	0
IX Circulatory	12	4 (3)	0	2 (1)	0	2 (0)	0	0	1 (0)	1 (0)	0
X Respiratory	4	3 (2)	0	0	0	0	0	0	1 (0)	0	0
XI Digestive	2	2 (2)	0	1 (1)	0	1 (0)	0	0	0	0	0
XIV Genitourinary	2	0	0	1 (0)	0	0	0	0	0	0	0
XVIII Other symptoms, signs and abnormal findings	2	1 (0)	0	0	0	0	0	0	1 (0)	0	0
XX External causes	5	2 (2)	0	1 (1)	0	0	0	0	0	0	0
Total	49	19 (14)	1 (1)	9 (3)	0	6 (0)	0	0	4 (1)	2 (0)	1 (1)

*Note:* The group with a BMI of 22.5–23.9 kg/m^2^ was the reference group. The numbers in brackets are the results after adjusting for false discovery rate (FDR).

Abbreviation: ICD‐10, International Classification of Diseases, 10th Revision.

After FDR adjustment, the underweight had a higher risk of dying at or after the age of 70 from seven diseases, whereas the other BMI groups had no statistically significant association with death risk from any outcome (Figure S2 and Table S6). Participants with underweight had an increased risk of dying at or after the age of 70 from stomach cancer (HR = 2.40, 95% CI: 1.56–3.71), less‐common neoplasms combined (2.63, 1.72–4.03), ischemic heart disease other than myocardial infarction (1.70, 1.33–2.17), myocardial infarction (1.50, 1.23–1.84), pneumonia (2.79, 1.74–4.45), COPD (2.18, 1.72–2.75) and falls (2.06, 1.32–3.20). The findings of sensitivity analyses are reported in the Supporting Information.

## 4 Discussion

This study was the first to examine the long‐term impacts of BMI, particularly underweight and low‐normal weight, on mortality risk from a wide range of diseases among Chinese female healthy never‐smokers. We found that underweight (BMI < 18.5 kg/m^2^) and low‐normal weight (18.5–19.9 and 20.0–22.4) were all associated with a higher mortality risk than BMI of 22.5–23.9, with association estimates for premature death higher than those for death at or after the age of 70. Underweight was linked to an increased risk of premature death in seven ICD‐10 chapters and 10 diseases, as well as an increased risk of death at or after the age of 70 in four chapters and seven diseases. In contrast, after adjusting for fat mass, obesity (≥ 28.0) was only associated with increased risks of premature death and death at or after the age of 70 in two and one ICD‐10 chapters, respectively.

A meta‐analysis of individual data summarized 141 prospective cohort studies involving 2.74 million women aged 20–89. Analyses excluded the first 5 years of follow‐up and restricted to never‐smokers without pre‐existing chronic disease, and found that individuals with a BMI of 15.0–18.5 kg/m^2^ had an HR of 1.53 (1.45–1.60), and all groups with a BMI above the reference range (18.5–25.0) were associated with an increased risk of all‐cause mortality, resulting in a J‐shaped relationship [6]. Another study included approximately 1.1 million participants aged ≥ 18 from 19 Asian cohorts. After adjusting for baseline comorbid conditions and excluding participants with < 2 years of follow‐up, among female never‐smokers in East Asia, the HRs for BMI < 18.5 and 18.5–19.9 were 1.55 (1.40–1.72) and 1.23 (1.11–1.36), respectively, compared to the reference group (BMI 23.0–24.9). The HRs for BMI of 30.0–31.9 and ≥ 32.0 were 1.18 (1.05–1.33) and 1.38 (1.24–1.53), respectively. Other BMI categories did not show statistically significant associations [8]. In contrast to previous findings, our study revealed an inverse J‐shaped relationship between BMI and all‐cause mortality risk, with the increased risk more pronounced at the lower extreme of BMI. This discrepancy may be partly explained by the lower prevalence of severe obesity among women in our cohort and the fact that most prior studies did not account for central obesity. We adjusted for WC for two primary reasons. First, this adjustment mitigates the confounding effect of central adiposity, allowing BMI to serve as a better proxy for lean body mass. Second, given the high prevalence of ‘normal weight central obesity’ in East Asian populations, without WC adjustment, abdominal fat accumulation within the reference BMI group (22.5–23.9 kg/m^2^) could artificially inflate baseline mortality risk and bias the results [24].

Only one study, based on the UK Clinical Practice Research Datalink (CPRD), investigated the relationship between BMI and 13 mid‐level disease categories and 28 specific causes of death, reporting association estimates for underweight and low‐normal weight. The study included men and women aged ≥ 16, with a mean age of 36.9 (26.6–52.4) and an average follow‐up of 12.7 (5.5) years [13]. The study excluded the first 5 years of follow‐up after the BMI record and limited the analysis to 1 969 648 non‐smokers. In contrast, our female population was older, had fewer obese participants and used different outcome definitions. As a result, the observed associations between BMI and death from diverse causes were not entirely consistent. A more detailed discussion will follow.

The UK CPRD study [13], and previous meta‐analyses conducted in European and American populations, which were restricted to non‐smokers [6, 7], found a J‐shaped relationship between BMI and cardiovascular mortality risk. Another study of East Asian populations found that the relationship between BMI and cardiovascular mortality among women was U‐shaped after excluding deaths within the first 3 years of follow‐up and adjusting for smoking status [25]. Our findings revealed that both underweight and obesity were associated with an increased risk of premature death from cardiovascular diseases and death at or after the age of 70, with the association being stronger for underweight than obesity and for premature than nonpremature death. In terms of specific causes of death, underweight was linked to an increased risk of dying from myocardial infarction, other ischemic heart diseases and intracerebral haemorrhage.

A study of 39 Asia‐Pacific cohorts investigated the association between BMI and cancer mortality risk among men and women aged ≥ 20, with a median follow‐up of 4 years and 4872 cancer deaths documented. After excluding participants with fewer than 3 years of follow‐up and adjusting for smoking, those who were underweight (< 18.5 kg/m^2^) or obese (≥ 30) had a modestly increased risk of cancer death [26]. The UK CPRD study only found an increase in cancer mortality risk among overweight and obese individuals; however, when specific types of cancer were evaluated, the risk of dying from oesophageal cancer increased among both underweight and overweight/obese individuals [13]. Our findings showed that both underweight and obese were associated with an increased risk of dying prematurely from cancer. After excluding those who died within the first 5 years of follow‐up, which resulted in a higher proportion of oesophageal, stomach and liver cancers excluded, the increased risk only persisted among obese women. The difference in the relationship between BMI and different cancer types, or undiagnosed cancer at baseline, may explain this change. In contrast, being underweight but not obese was associated with an increased risk of dying from cancer at or after the age of 70, particularly stomach cancer and less‐common neoplasms combined. The results were robust even when deaths within the first 5 years were excluded.

Among UK CPRD non‐smokers, the association between BMI and respiratory mortality risk was approximately U‐shaped, with a similar relationship observed for lower respiratory infection [13]. The aforementioned analysis of 19 Asian cohorts found that among non‐smoking East Asian women, both low BMI and obesity were associated with an increased mortality risk from respiratory diseases [8]. A Korean study of over 410 000 women aged 30–95, who were non‐smokers and free of major diseases at baseline, found an increased risk of dying from respiratory diseases among those with low BMI (< 20 kg/m^2^) but not overweight or obese. Such an association was also observed for the risk of mortality from major lung diseases (including tuberculosis, COPD, asthma and pneumonia), even after excluding the first 5 years of follow‐up [27]. Our findings showed that women who were underweight or had a low‐normal weight were more likely to die from respiratory system diseases. More specifically, underweight was linked to both premature and nonpremature death from COPD, but only death from pneumonia at or after the age of 70.

After adjusting for baseline diabetes status, the UK CPRD study indicated that individuals with underweight (< 18.5 kg/m^2^) and obese (> 30) had a higher mortality risk from blood and endocrine diseases (ICD‐10 chapter: D50‐89, E) than those with normal weight (18.5–25.0) [13]. A recent Korean nationwide cohort study of nearly 1.8 million adults with type 2 diabetes reported a dose–response relationship between severity of underweight and diabetes‐related mortality. Adjusted HRs (95% CI) ranged from 2.265 (95% CI: 2.073–2.474) for mild underweight to 5.136 (95% CI: 4.300–6.134) for severe underweight, with risks exceeding those observed in severely obese individuals [28]. Notably, we found an association between underweight and an increased mortality risk in the endocrine‐metabolic chapter. After excluding women with baseline diabetes, obese individuals also showed an increased risk of dying prematurely from this chapter's diseases, whereas the HR for those who were underweight increased. This could be explained by the fact that not excluding baseline diabetic patients may lead to an overestimation of the reference group risk. Furthermore, even after excluding those with baseline diabetes or the first 5 years of follow‐up, underweight was persistently associated with an increased risk of dying from type 2 diabetes. Previous studies in European and American populations only linked obesity to an increased risk of diabetes‐related death [11, 13], presumably due to the small number of underweight participants. Underweight individuals generally have poorer overall health and nutritional status, predisposing them to more severe metabolic disorders, accelerated progression of complications and compromised immune function [14]. Furthermore, in some underweight individuals with type 2 diabetes, a sarcopenic phenotype characterized by reduced skeletal muscle mass may further impair glucose metabolism, increase insulin resistance, worsen glycemic control and elevate mortality risk [28].

In the UK CPRD study, BMI had a J‐shaped relationship with the risk of dying from digestive (excluding cirrhosis) and urogenital diseases. Only low BMI was associated with an increased risk of death from accidents (excluding transport) and specific causes of death such as falls [13]. However, in our population, after adjusting for WC, only the underweight group had an increased risk of death from digestive, genitourinary and external causes. Similarly, a Swiss study included 31 578 men and women aged 25–74, with women accounting for nearly 90% of the underweight individuals and a mean follow‐up period of 20 years. After adjusting for smoking status, underweight was associated with an increased risk of death from external causes, which was explained by the fact that underweight not only increases the risk of accidental injuries but also reduces the probability of survival after incidents [29].

Our study has several strengths. Due to the large sample size and long follow‐up time, a substantial number of deaths were observed. This provided us with sufficient power to use detailed BMI classifications, focus on those with underweight and low‐normal weight, distinguish between premature and nonpremature death and apply a unified statistical strategy within the same population to compare the relationship between BMI and mortality from a wide range of diseases. We maximized bias control by limiting analyses to never‐smokers, excluding participants with major illnesses at baseline and removing the first 5 years of follow‐up. Height, weight and WC were measured on‐site rather than self‐reported, and there was virtually no missing data for these variables and other covariates.

The study also has limitations. First, we only used baseline BMI without considering its changes over time. However, incorporating follow‐up BMI measurements may introduce reverse causation bias due to weight loss resulting from incident diseases. Furthermore, the natural decrease in height associated with ageing could lead to an underestimation of BMI in later measurements. Second, our analysis was restricted to women. Sex differences in the BMI–mortality associations are plausible given differences in body composition, fat distribution, hormonal profiles and musculoskeletal health. Future analyses including men would be valuable once the follow‐up period is extended and a sufficient number of events have occurred. Third, because we only collected medical histories for around 20 common diseases, we were unable to exclude all relevant diseases at baseline from the cause‐specific analysis, which limited the interpretation of the results to some extent. Fourth, due to the nature of the observational study design, caution should be exercised when inferring causation.

## 5 Conclusions

In this study of nearly 300 000 Chinese women, underweight was an important risk factor for all‐cause and multiple cause‐specific mortality. Furthermore, the association strength of underweight with premature death was stronger than that with death at or after the age of 70. There were also differences in the specific causes of death related to underweight between premature and nonpremature death. Weight is an important determinant of the body's metabolic, reproductive, immunological and musculoskeletal health, and both low and high weight can be harmful. While addressing the global obesity epidemic, the negative health consequences of low weight should not be ignored. Given the dual challenges facing developing countries, the message for population‐wide campaigns should prioritize maintaining a healthy weight, or losing fat while gaining muscle, above losing weight.

## Funding

This work was supported by National Natural Science Foundation of China (82388102, 82192901, 82192904 and 82192900) and the Noncommunicable Chronic Diseases‐National Science and Technology Major Project (2023ZD0510101 and 2023ZD0510100). The CKB baseline survey and the first resurvey were supported by the Kadoorie Charitable Foundation in Hong Kong. The long‐term follow‐up has been supported by Wellcome grants to Oxford University (212946/Z/18/Z, 202922/Z/16/Z, 104085/Z/14/Z and 088158/Z/09/Z) and grants (2016YFC0900500) from the National Key R&D Program of China, National Natural Science Foundation of China (81390540, 91846303 and 81941018) and Chinese Ministry of Science and Technology (2011BAI09B01). The UK Medical Research Council (MC_UU_00017/1, MC_UU_12026/2 and MC_U137686851), Cancer Research UK (C16077/A29186 and C500/A16896) and the British Heart Foundation (CH/1996001/9454) provide core funding to the Clinical Trial Service Unit and Epidemiological Studies Unit at Oxford University for the project.

## Conflicts of Interest

The authors declare no conflicts of interest.

## Supporting information


**Table S1:** Cause‐specific mortality outcome classification and ICD‐10 codes.
**Table S2:** Primary and sensitivity analyses of the associations between BMI and all‐cause mortality.
**Table S3:** Associations between BMI and ICD‐10 chapter‐specific mortality.
**Table S4:** Associations between BMI and ICD‐10 chapter‐specific mortality after excluding those with relevant pre‐existing diseases reported at baseline.
**Table S5:** Associations between BMI and ICD‐10 chapter‐specific mortality after excluding those who died within the first 5 years of follow‐up.
**Table S6:** Associations between BMI and cause‐specific mortality.
**Table S7:** Summary of number of diseases associated with BMI by ICD‐10 chapter after excluding those with relevant pre‐existing diseases reported at baseline.
**Table S8:** Summary of number of diseases associated with BMI by ICD‐10 chapter after excluding those who died within the first 5 years of follow‐up.
**Table S9:** Associations between BMI and cause‐specific mortality after excluding those with relevant pre‐existing diseases reported at baseline.
**Table S10:** Associations between BMI and cause‐specific mortality after excluding those who died within the first 5 years of follow‐up.
**Figure S1:** The flow chart of participants.
**Figure S2:** Adjusted HRs for cause‐specific death at or after the age of 70 associated with body mass index.

## Data Availability

Details of how to access China Kadoorie Biobank data and details of the data release schedule are available from www.ckbiobank.org/site/Data+Access.
