# Supplementary material for: Body Mass Index and Risks of More Than 40 Cause‐Specific Mortality in Chinese Women: A Prospective Cohort Study
Source: J Cachexia Sarcopenia Muscle. 2026 Jul 16;17(4):e70330. doi: 10.1002/jcsm.70330 (PMC13374634; doi:10.1002/jcsm.70330)
Supplement: Supplementary file 1 — Table S1: Cause‐specific mortality outcome classification and ICD‐10 codes. Table S2: Primary and sensitivity analyses of the associations between BMI and all‐cause mortality. Table S3: Associations between BMI and ICD‐10 chapter‐specific mortality. Table S4: Associations between BMI and ICD‐10 chapter‐specific mortality after excluding those with relevant pre‐existing diseases reported at baseline. Table S5: Associations between BMI and ICD‐10 chapter‐specific mortality after excluding those who died within the first 5 years of follow‐up. Table S6: Associations between BMI and cause‐specific mortality. Table S7: Summary of number of diseases associated with BMI by ICD‐10 chapter after excluding those with relevant pre‐existing diseases reported at baseline. Table S8: Summary of number of diseases associated with BMI by ICD‐10 chapter after excluding those who died within the first 5 years of follow‐up. Table S9: Associations between BMI and cause‐specific mortality after excluding those with relevant pre‐existing diseases reported at baseline. Table S10: Associations between BMI and cause‐specific mortality after excluding those who died within the first 5 years of follow‐up. Figure S1: The flow chart of participants. Figure S2: Adjusted HRs for cause‐specific death at or after the age of 70 associated with body mass index. [file JCSM-17-e70330-s001.docx]

Supplemental material for the manuscript entitled **Body mass index and risks of more than 40 cause-specific mortality in Chinese women: a prospective cohort study**

**Contents**

[Members of the China Kadoorie Biobank collaborative group 2](#_Toc224135547)

[Results of sensitivity analyses 3](#_Toc224135548)

[Table S1. Cause-specific mortality outcome classification and ICD-10 codes 5](#_Toc224135549)

[Table S2. Primary and sensitivity analyses of the associations between BMI and all-cause mortality 7](#_Toc224135550)

[Table S3. Associations between BMI and ICD-10 chapter-specific mortality 8](#_Toc224135551)

[Table S4. Associations between BMI and ICD-10 chapter-specific mortality after excluding those with relevant pre-existing diseases reported at baseline 11](#_Toc224135552)

[Table S5. Associations between BMI and ICD-10 chapter-specific mortality after excluding those who died within the first five years of follow-up 12](#_Toc224135553)

[Table S6. Associations between BMI and cause-specific mortality 15](#_Toc224135554)

[Table S7. Summary of number of diseases associated with BMI by ICD-10 chapter after excluding those with relevant pre-existing diseases reported at baseline 24](#_Toc224135555)

[Table S8. Summary of number of diseases associated with BMI by ICD-10 chapter after excluding those who died within the first five years of follow-up 26](#_Toc224135556)

[Table S9. Associations between BMI and cause-specific mortality after excluding those with relevant pre-existing diseases reported at baseline 28](#_Toc224135557)

[Table S10. Associations between BMI and cause-specific mortality after excluding those who died within the first five years of follow-up 30](#_Toc224135558)

[Figure S1. The flowchart of participants 39](#_Toc224135559)

[Figure S2. Adjusted HRs for cause-specific death at or after the age of 70 associated with body mass index 40](#_Toc224135560)

**Members of the China Kadoorie Biobank collaborative group**

**International Steering Committee:** Junshi Chen, Zhengming Chen (PI), Robert Clarke, Rory Collins, Liming Li (PI), Jun Lv, Richard Peto, Robin Walters.

**International Co-ordinating Centre, Oxford:** Daniel Avery, Maxim Barnard, Derrick Bennett, Ruth Boxall, Ka Hung Chan, Yiping Chen, Zhengming Chen, Charlotte Clarke, Johnathan Clarke; Robert Clarke, Huaidong Du, Ahmed Edris Mohamed, Hannah Fry, Simon Gilbert, Pek Kei Im, Andri Iona, Maria Kakkoura, Christiana Kartsonaki, Kshitij Kolhe, Hubert Lam, Kuang Lin, James Liu, Mohsen Mazidi, Iona Millwood, Sam Morris, Qunhua Nie, Alfred Pozarickij, Maryam Rahmati, Paul Ryder, Dan Schmidt, Becky Stevens, Iain Turnbull, Robin Walters, Baihan Wang, Lin Wang, Neil Wright, Ling Yang, Xiaoming Yang, Pang Yao.

**National Co-ordinating Centre, Beijing:** Jun Lv, Canqing Yu, Dianjianyi Sun, Yuanjie Pang, Can Hou, Qingmei Xia, Chao Liu, Pei Pei, Lang Pan, Xiao Han, Honglu Bian, Xinxin Chen.

**10 Regional Co-ordinating Centres:**

**Qingdao CDC:** Zengchang Pang, Ruqin Gao, Shanpeng Li, Haiping Duan, Shaojie Wang, Yongmei Liu, Ranran Du, Liang Cheng, Xiaocao Tian, Hua Zhang. **Licang CDC:** Dan Hu, Xiaoyan Zheng, Yujie Wang. **Heilongjiang Provincial CDC:** Wei Sun, Shichun Yan, Xiaoming Cui. **Nangang CDC:** Chi Wang, Zhenyuan Wu, Lishun Zhai, Zhaoxi Pang, Shiwen Dong. **Hainan Provincial CDC:** Huiming Luo, Jinyan Chen, Bin He, Dingwei Sun, Xingren Wang, Tingting Ou. **Meilan CDC:** Xiangyang Zheng, Dewei Zheng, Shuai Yang, Yilei Li, Lihui Li, Xingjiao Chen. **Jiangsu Provincial CDC:** Jinyi Zhou, Ran Tao, Jian Su, Xikang Fan, Zongming Cheng, Yuxiao Huang. **Suzhou CDC:** Yan Lu, Yujie Hua, Li Xing, Shuxian Wang, Jianrong Jin, Juping Ma, Jinchao Liu, Kaifei Zhu, Hongfu Ren, Xingfeng Shen. **Guangxi Provincial CDC:** Ge Zhong, Wei Mao, Zhenzhen Lu, Ling He. **Liuzhou CDC:** Lifang Zhou, Changping Xie, Jian Lan, Tingping Zhu, Jinxue Tan, Liuping Wei, Liyuan Zhou, Sisi Wang. **Sichuan Provincial CDC:** Xianping Wu, Ningmei Zhang, Xiaofang Chen, Xiaoyu Chang, Zhuo Wang, Yujin He. **Pengzhou CDC:** Mingqiang Yuan, Xia Wu, Xiaofang Chen, Zhaodong Wang, Qiang Sun, Yang Lin. **Gansu Provincial CDC:** Faqing Chen, Xiaolan Ren, Lijun Chang, Feiming Zhong. **Maiji CDC:** Jianjun Feng, Weijie Hu, Xiaofang Zhang, Yalin Chen, Fei Wang, Jun Wang. **Henan Provincial CDC:** Linqi Diao, Wanshen Guo, Zhiwei Han, Dongyang Zhao, Dengjun Zhu, Kai Kang, Shixian Feng, Huizi Tian, Yali Yan, Bing Han, Li Gao, Shaofang Li, Huafei Feng, Wei Tang. **Huixian CDC:** Xiaolin Li, Huarong Sun, Xiaocong Zhao, Ying Li, Chen Hu, Pan He, Xukui Zhang, Yuanyuan Jin, Hesheng Zhang. **Zhejiang Provincial CDC:** Min Yu, Ruying Hu, Hao Wang, Weiwei Gong, Jieming Zhong, Meng Wang, Chunxiao Xu, Keqing Gong. **Tongxiang CDC**: Hao Xu, Yuan Cao, Kaixu Xie, Lingli Chen, Xiaomei Tu,Chen Chen. **Hunan Provincial CDC:** Xiaojun Li, Li Yin, Huilin Liu, Yuan Liu, Yi Liu, Lei Yin, Xian Xie, Jing Wang. **Liuyang CDC:** Bo Xiao, Pingsheng Lou, Yuan Peng, Libo Zhang, Chan Qu, Qili Jiang, Yanling Chen, Yan Zhao.

**Results of sensitivity analyses**

**Association between BMI and all-cause mortality**

After excluding those with relevant pre-existing diseases reported at baseline or those who died within the first five years of follow-up, the HRs for premature death in the three BMI groups below the reference group were slightly attenuated (Supplementary Table 2). Furthermore, after excluding those with relevant pre-existing diseases reported at baseline, obesity was associated with an increased risk of premature death.

**Association between BMI and chapter-specific mortality**

After excluding those with relevant pre-existing diseases reported at baseline, the association between underweight and each chapter remained largely consistent; however, for the other two groups below the reference group, some associations disappeared (Supplementary Table 4). After excluding participants with diabetes at baseline, obesity was linked to an increased risk of premature death from endocrine-metabolic diseases. After excluding those who died within the first five years of follow-up, underweight was no longer statistically significant associated with the risk of premature death from neoplasms, genitourinary, and external causes (Supplementary Table 5).

**Association between BMI and cause-specific mortality**

After excluding those with relevant pre-existing diseases reported at baseline (Supplementary Table 7) or those who died within the first five years of follow-up (Supplementary Table 8), the number of outcomes that could be analyzed decreased. The number of causes of premature death linked to underweight reduced to 8 and 4 in the above two sensitivity analyses, respectively, while the results for death at or after the age of 70 did not change significantly. After excluding participants with diabetes at baseline, a BMI of 18.5-19.9 kg/m^2^ was no longer linked to the risk of premature death from type 2 diabetes; however, overweight and obesity exhibited a positive association (Supplementary Table 9). After excluding those who died within the first five years of follow-up, being underweight still had an increased risk of premature death from four diseases: type 2 diabetes, other ischemic heart disease, myocardial infarction, and COPD (Supplementary Table 10).

**Table S1. Cause-specific mortality outcome classification and ICD-10 codes**

| **Outcome** | **ICD-10 codes** |
| --- | --- |
| **I Certain infectious and parasitic diseases** | **A00-B99** |
| Viral hepatitis | B15-B19 |
| Less-common certain infectious and parasitic diseases combined^*^ | Rest of A00-B99 |
| **II Neoplasms** | **C00-D48** |
| Malignant neoplasms of lip, oral cavity and pharynx | C00-C14 |
| Malignant neoplasm of oesophagus | C15 |
| Malignant neoplasm of stomach | C16 |
| Malignant neoplasm of colon and rectum | C18-C20 |
| Malignant neoplasm of liver and intrahepatic bile ducts | C22 |
| Malignant neoplasm of gallbladder and biliary tract | C23-C24 |
| Malignant neoplasm of pancreas | C25 |
| Malignant neoplasm of trachea, bronchus and lung | C33-C34 |
| Malignant neoplasm of breast | C50 |
| Malignant neoplasm of cervix uteri | C53 |
| Malignant neoplasm of corpus uteri | C54 |
| Malignant neoplasm of uterus, part unspecified | C55 |
| Malignant neoplasm of ovary | C56 |
| Malignant neoplasms of central nervous system | C70-C72 |
| Malignant neoplasms, stated or presumed to be primary, of lymphoid, haematopoietic and related tissue | C81-C96 |
| Less-common neoplasms combined^*^ | Rest of C00-D48 |
| **IV Endocrine, nutritional and metabolic diseases** | **E00-E90** |
| Type 2 diabetes mellitus | E11, E14 |
| Less-common endocrine, nutritional and metabolic diseases combined^*^ | Rest of E00-E90 |
| **V Mental and behavioural disorders** | **F00-F99** |
| **VI Diseases of the nervous system** | **G00-G99** |
| Other degenerative diseases of nervous system, not elsewhere classified | G31 |
| Less-common diseases of the nervous system combined^*^ | Rest of G00-G99 |
| **IX Diseases of the circulatory system** | **I00-I99** |
| Chronic rheumatic heart diseases | I05-I09 |
| Hypertensive diseases | I10-I15 |
| Ischaemic heart diseases other than myocardial infarction | I20, I24, I25 |
| Myocardial infarction | I21-I23 |
| Cardiac arrest | I46 |
| Subarachnoid haemorrhage | I60 |
| Intracerebral haemorrhage | I61 |
| Cerebral infarction | I63 |
| Stroke, not specified as haemorrhage or infarction | I64 |
| Other cerebrovascular diseases | I67 |
| Sequelae of cerebrovascular disease | I69 |
| Less-common diseases of the circulatory system combined^*^ | Rest of I00-I99 |
| **X Diseases of the respiratory system** | **J00-J99** |
| Pneumonia | J12-J18 |
| Chronic obstructive pulmonary disease | J41-J44 |
| Other respiratory disorders | J98 |
| Less-common diseases of the respiratory system combined^*^ | Rest of J00-J99 |
| **XI Diseases of the digestive system** | **K00-K93** |
| Diseases of liver | K70-K77 |
| Less-common diseases of the digestive system combined^*^ | Rest of K00-K93 |
| **XIII Diseases of the musculoskeletal system and connective tissue** | **M00-M99** |
| **XIV Diseases of the genitourinary system** | **N00-N99** |
| Chronic nephritic syndrome | N03 |
| Less-common diseases of the genitourinary system combined^*^ | Rest of N00-N99 |
| **XVIII Symptoms, signs and abnormal clinical and laboratory findings, not elsewhere classified** | R00-R99 |
| Senility | R54 |
| Ill-defined and unknown causes of mortality | R96-R99 |
| **XX External causes of morbidity and mortality** | **V01-Y98** |
| Transport accidents | V01-V99 |
| Falls | W00-W19 |
| Intentional self-harm | X60-X84 |
| Sequelae of other accidents | Y86 |
| Less-common external causes of morbidity and mortality combined^*^ | Rest of V01-Y98 |
| **All causes** | **A00-Y98** |

ICD-10, International Classification of Diseases, 10th Revision.

^*^Included less-common ICD-10 codes within the corresponding ICD-10 chapter that were not individually investigated in the present study.

**Table S2. Primary and sensitivity analyses of the associations between BMI and all-cause mortality**

|  | **No. of deaths** | **<18.5 kg/m^2^** | **18.5-19.9 kg/m^2^** | **20.0-22.4 kg/m^2^** | **22.5-23.9 kg/m^2^** | **24.0-27.9 kg/m^2^** | **≥28.0 kg/m^2^** | ***P*_trend_** |
| --- | --- | --- | --- | --- | --- | --- | --- | --- |
| **Primary analysis (N=262,704)** | | | | | | | | |
| Premature death | 11,455 | **1.91 (1.73-2.10)** | **1.24 (1.14-1.34)** | **1.16 (1.11-1.21)** | 1.00 (0.96-1.04) | 0.99 (0.95-1.03) | 1.04 (0.97-1.12) | <0.001 |
| Death at or after the age of 70 | 18,076 | **1.46 (1.36-1.56)** | **1.17 (1.10-1.23)** | 1.04 (1.01-1.08) | 1.00 (0.97-1.04) | 0.97 (0.94-1.00) | 1.03 (0.98-1.10) | <0.001 |
| All death | 29,531 | **1.60 (1.51-1.69)** | **1.20 (1.15-1.26)** | **1.09 (1.06-1.12)** | 1.00 (0.97-1.03) | 0.98 (0.95-1.00) | 1.04 (0.99-1.09) | <0.001 |
| **Excluded participants with other known diseases at baseline^*^ (N=219,230)** | | | | | | | | |
| Premature death | 8,585 | **1.71 (1.53-1.91)** | 1.10 (1.00-1.20) | **1.12 (1.06-1.17)** | 1.00 (0.95-1.05) | 1.04 (0.99-1.08) | **1.14 (1.05-1.24)** | 0.061 |
| Death at or after the age of 70 | 12,980 | **1.43 (1.32-1.54)** | **1.12 (1.05-1.19)** | 1.04 (1.00-1.08) | 1.00 (0.96-1.04) | 0.97 (0.93-1.01) | 1.07 (1.00-1.15) | 0.005 |
| All death | 21,565 | **1.52 (1.42-1.62)** | **1.12 (1.06-1.18)** | **1.07 (1.04-1.11)** | 1.00 (0.97-1.03) | 1.00 (0.97-1.03) | **1.10 (1.04-1.16)** | 0.001 |
| **Excluded participants who died within the first five years of follow-up (N=258,336)** | | | | | | | | |
| Premature death | 8,519 | **1.62 (1.44-1.82)** | **1.12 (1.02-1.23)** | **1.13 (1.07-1.19)** | 1.00 (0.95-1.05) | 0.98 (0.93-1.02) | 1.10 (1.01-1.19) | 0.009 |
| Death at or after the age of 70 | 16,644 | **1.46 (1.36-1.57)** | **1.16 (1.09-1.23)** | 1.03 (0.99-1.07) | 1.00 (0.96-1.04) | 0.96 (0.93-1.00) | 1.03 (0.97-1.09) | <0.001 |
| All death | 25,163 | **1.51 (1.42-1.60)** | **1.15 (1.10-1.21)** | **1.06 (1.03-1.10)** | 1.00 (0.97-1.03) | 0.97 (0.94-1.00) | 1.05 (1.00-1.11) | <0.001 |

The models were adjusted for the same covariates as described in Figure 1. Linear trend tests were performed with the median value within each body mass index group. Bold indicates *P*<0.05.

^*^Other known diseases include diabetes, tuberculosis, hepatitis/cirrhosis, peptic ulcer, gallstones/gallbladder disease, and chronic kidney disease.

**Table S3. Associations between BMI and ICD-10 chapter-specific mortality**

| **Chapter** | **No. of deaths** | **<18.5 kg/m^2^** | **18.5-19.9 kg/m^2^** | **20.0-22.4 kg/m^2^** | **22.5-23.9 kg/m^2^** | **24.0-27.9 kg/m^2^** | **≥28.0 kg/m^2^** | ***P*_trend_** |
| --- | --- | --- | --- | --- | --- | --- | --- | --- |
| **I Certain infectious and parasitic diseases** | | | | | | | | |
| Premature death | 161 | 1.16 (0.51-2.61) | 0.67 (0.33-1.36) | 1.00 (0.71-1.41) | 1.00 (0.72-1.39) | 0.80 (0.56-1.14) | 0.88 (0.46-1.69) | 0.769 |
| Death at or after the age of 70 | 106 | 2.49 (1.16-5.37) | 1.79 (0.95-3.38) | 1.06 (0.68-1.63) | 1.00 (0.60-1.67) | 1.23 (0.77-1.97) | 1.12 (0.48-2.63) | 0.439 |
| All death | 267 | 1.63 (0.94-2.82) | 1.04 (0.65-1.67) | 1.02 (0.78-1.33) | 1.00 (0.76-1.32) | 0.93 (0.70-1.24) | 0.94 (0.56-1.59) | 0.443 |
| **II Neoplasms** | | | | | | | | |
| Premature death | 4,830 | **1.36 (1.16-1.59)** | 1.10 (0.98-1.24) | **1.17 (1.09-1.24)** | 1.00 (0.94-1.07) | 1.07 (1.01-1.14) | **1.15 (1.03-1.29)** | 0.503 |
| Death at or after the age of 70 | 3,857 | **1.45 (1.24-1.70)** | 1.06 (0.93-1.21) | 1.06 (0.98-1.14) | 1.00 (0.93-1.08) | 1.01 (0.94-1.08) | 1.04 (0.92-1.17) | 0.183 |
| All death | 8,687 | **1.41 (1.26-1.57)** | 1.09 (0.99-1.19) | **1.12 (1.07-1.18)** | 1.00 (0.95-1.05) | 1.04 (1.00-1.09) | 1.10 (1.01-1.19) | 0.162 |
| **IV Endocrine, nutritional and metabolic diseases** | | | | | | | | |
| Premature death | 492 | **6.03 (4.13-8.82)** | **3.08 (2.25-4.21)** | 1.21 (0.98-1.50) | 1.00 (0.81-1.24) | **0.62 (0.50-0.75)** | **0.45 (0.31-0.64)** | <0.001 |
| Death at or after the age of 70 | 864 | **1.56 (1.07-2.27)** | 0.88 (0.63-1.24) | 1.17 (0.99-1.38) | 1.00 (0.85-1.17) | 0.81 (0.71-0.93) | **0.69 (0.54-0.87)** | 0.002 |
| All death | 1,356 | **2.67 (2.04-3.50)** | **1.52 (1.21-1.91)** | 1.19 (1.04-1.36) | 1.00 (0.88-1.14) | **0.73 (0.66-0.82)** | **0.58 (0.48-0.71)** | <0.001 |
| **V Mental and behavioural disorders** | | | | | | | | |
| Premature death | / | / | / | / | / | / | / | / |
| Death at or after the age of 70 | 136 | 1.58 (0.79-3.17) | 1.80 (1.08-3.00) | 1.09 (0.78-1.52) | 1.00 (0.65-1.54) | 0.98 (0.63-1.54) | 0.56 (0.23-1.39) | 0.096 |
| All death | 166 | 1.60 (0.81-3.15) | 1.76 (1.07-2.88) | 1.13 (0.83-1.55) | 1.00 (0.69-1.46) | 0.96 (0.65-1.41) | 0.69 (0.33-1.43) | 0.099 |
| **VI Diseases of the nervous system** | | | | | | | | |
| Premature death | 110 | 1.76 (0.73-4.24) | 1.37 (0.70-2.68) | 1.22 (0.84-1.79) | 1.00 (0.62-1.61) | 1.49 (0.94-2.35) | 0.81 (0.29-2.24) | 0.690 |
| Death at or after the age of 70 | 275 | 1.02 (0.62-1.66) | 1.10 (0.75-1.62) | 0.97 (0.77-1.22) | 1.00 (0.75-1.34) | 1.01 (0.73-1.38) | 1.12 (0.63-2.01) | 0.932 |
| All death | 385 | 1.17 (0.76-1.79) | 1.18 (0.84-1.64) | 1.04 (0.86-1.27) | 1.00 (0.78-1.28) | 1.15 (0.89-1.48) | 1.06 (0.64-1.75) | 0.944 |
| **IX Diseases of the circulatory system** | | | | | | | | |
| Premature death | 3,767 | **1.85 (1.56-2.20)** | 1.10 (0.95-1.27) | 1.12 (1.03-1.21) | 1.00 (0.92-1.08) | 0.99 (0.93-1.06) | **1.17 (1.04-1.31)** | 0.254 |
| Death at or after the age of 70 | 9,377 | **1.30 (1.18-1.43)** | **1.16 (1.07-1.25)** | 1.03 (0.98-1.08) | 1.00 (0.95-1.05) | 1.01 (0.97-1.06) | **1.11 (1.03-1.20)** | 0.404 |
| All death | 13,144 | **1.43 (1.31-1.56)** | **1.16 (1.08-1.24)** | 1.05 (1.01-1.10) | 1.00 (0.96-1.04) | 1.01 (0.97-1.05) | **1.14 (1.06-1.21)** | 0.193 |
| **X Diseases of the respiratory system** | | | | | | | | |
| Premature death | 342 | **5.85 (3.89-8.80)** | **1.87 (1.24-2.81)** | **1.56 (1.25-1.95)** | 1.00 (0.75-1.34) | 1.03 (0.79-1.34) | 1.10 (0.69-1.75) | <0.001 |
| Death at or after the age of 70 | 1,464 | **2.44 (2.00-2.97)** | **1.28 (1.07-1.54)** | 1.10 (0.98-1.23) | 1.00 (0.88-1.14) | 0.86 (0.75-0.98) | 1.00 (0.80-1.25) | <0.001 |
| All death | 1,806 | **2.84 (2.37-3.40)** | **1.38 (1.17-1.63)** | **1.18 (1.07-1.30)** | 1.00 (0.89-1.13) | 0.89 (0.79-1.00) | 1.01 (0.83-1.24) | <0.001 |
| **XI Diseases of the digestive system** | | | | | | | | |
| Premature death | 198 | **5.64 (3.13-10.18)** | **1.98 (1.13-3.45)** | **1.69 (1.28-2.24)** | 1.00 (0.71-1.41) | 0.69 (0.49-0.97) | **0.47 (0.25-0.90)** | <0.001 |
| Death at or after the age of 70 | 368 | 1.53 (0.90-2.59) | 1.48 (1.00-2.20) | 1.31 (1.04-1.65) | 1.00 (0.78-1.29) | 0.90 (0.71-1.13) | 0.82 (0.54-1.25) | 0.040 |
| All death | 566 | **2.48 (1.67-3.69)** | **1.67 (1.21-2.30)** | **1.45 (1.22-1.73)** | 1.00 (0.82-1.22) | 0.82 (0.68-1.00) | 0.69 (0.49-0.98) | <0.001 |
| **XIII Diseases of the musculoskeletal system and connective tissue** | | | | | | | | |
| Premature death | / | / | / | / | / | / | / | / |
| Death at or after the age of 70 | / | / | / | / | / | / | / | / |
| All death | 115 | 1.32 (0.57-3.05) | 1.22 (0.64-2.31) | 0.89 (0.59-1.32) | 1.00 (0.66-1.51) | 0.77 (0.49-1.23) | 1.40 (0.65-3.01) | 0.965 |
| **XIV Diseases of the genitourinary system** | | | | | | | | |
| Premature death | 138 | **2.61 (1.20-5.69)** | 1.98 (1.10-3.55) | 1.10 (0.76-1.60) | 1.00 (0.69-1.45) | 0.63 (0.42-0.95) | 0.68 (0.34-1.37) | 0.016 |
| Death at or after the age of 70 | 183 | 1.78 (0.88-3.63) | 1.50 (0.85-2.64) | 1.21 (0.87-1.70) | 1.00 (0.70-1.44) | 1.00 (0.72-1.39) | 0.83 (0.46-1.52) | 0.187 |
| All death | 321 | **2.13 (1.26-3.61)** | **1.71 (1.14-2.57)** | 1.16 (0.91-1.49) | 1.00 (0.77-1.30) | 0.82 (0.64-1.06) | 0.76 (0.48-1.20) | 0.011 |
| **XVIII Symptoms, signs and abnormal clinical and laboratory findings, not elsewhere classified** | | | | | | | | |
| Premature death | 206 | 2.01 (0.99-4.06) | 1.44 (0.84-2.48) | 0.97 (0.69-1.34) | 1.00 (0.74-1.36) | 0.86 (0.64-1.15) | 0.86 (0.49-1.49) | 0.203 |
| Death at or after the age of 70 | 426 | 1.36 (0.86-2.15) | 1.26 (0.88-1.81) | 0.97 (0.78-1.22) | 1.00 (0.81-1.24) | 0.78 (0.63-0.97) | 0.85 (0.58-1.25) | 0.130 |
| All death | 632 | 1.52 (1.03-2.24) | 1.31 (0.97-1.77) | 0.97 (0.81-1.17) | 1.00 (0.84-1.19) | 0.81 (0.68-0.96) | 0.86 (0.63-1.17) | 0.053 |
| **XX External causes of morbidity and mortality** | | | | | | | | |
| Premature death | 1,053 | **2.06 (1.55-2.72)** | 1.29 (1.03-1.62) | 1.06 (0.93-1.20) | 1.00 (0.87-1.15) | 1.02 (0.88-1.18) | 0.76 (0.57-1.01) | 0.002 |
| Death at or after the age of 70 | 843 | 1.27 (0.95-1.69) | 1.21 (0.97-1.52) | 0.96 (0.83-1.10) | 1.00 (0.85-1.18) | 0.88 (0.74-1.04) | 1.03 (0.76-1.39) | 0.284 |
| All death | 1,896 | **1.62 (1.32-1.98)** | **1.27 (1.08-1.49)** | 1.02 (0.92-1.12) | 1.00 (0.90-1.11) | 0.96 (0.86-1.07) | 0.87 (0.71-1.07) | 0.003 |

ICD-10, International Classification of Diseases, 10th Revision.

The models were adjusted for the same covariates as described in Figure 1. Linear trend tests were performed with the median value within each body mass index group. Bold indicates *P*<0.05.

**Table S4. Associations between BMI and ICD-10 chapter-specific mortality after excluding those with relevant pre-existing diseases reported at baseline**

| **Chapter** | **No. of deaths** | **<18.5 kg/m^2^** | **18.5-19.9 kg/m^2^** | **20.0-22.4 kg/m^2^** | **22.5-23.9 kg/m^2^** | **24.0-27.9 kg/m^2^** | **≥28.0 kg/m^2^** | ***P*_trend_** |
| --- | --- | --- | --- | --- | --- | --- | --- | --- |
| **I Certain infectious and parasitic diseases (****excluding 4,586 participants with tuberculosis or hepatitis/cirrhosis at baseline)** | | | | | | | | |
| Premature death | 145 | 0.92 (0.35-2.47) | 0.81 (0.39-1.67) | 1.07 (0.74-1.54) | 1.00 (0.71-1.42) | 0.82 (0.57-1.17) | 0.89 (0.46-1.76) | 0.748 |
| Death at or after the age of 70 | 104 | 2.49 (1.14-5.46) | 1.88 (0.99-3.55) | 1.08 (0.70-1.67) | 1.00 (0.60-1.67) | 1.17 (0.72-1.88) | 1.05 (0.44-2.47) | 0.347 |
| All death | 249 | 1.57 (0.87-2.83) | 1.19 (0.74-1.92) | 1.07 (0.81-1.41) | 1.00 (0.75-1.34) | 0.93 (0.70-1.25) | 0.94 (0.55-1.60) | 0.369 |
| **IV Endocrine, nutritional and metabolic diseases (excluding 14,281 participants with diabetes at baseline)** | | | | | | | | |
| Premature death | 128 | **7.12 (3.13-16.19)** | 2.59 (1.18-5.66) | 1.52 (0.92-2.51) | 1.00 (0.56-1.80) | 1.58 (1.15-2.16) | **2.65 (1.52-4.63)** | 0.821 |
| Death at or after the age of 70 | 356 | 1.61 (0.92-2.84) | 0.82 (0.48-1.41) | 1.07 (0.81-1.41) | 1.00 (0.77-1.30) | 0.96 (0.78-1.17) | 1.09 (0.76-1.56) | 0.943 |
| All death | 484 | **2.32 (1.45-3.70)** | 1.10 (0.71-1.71) | 1.15 (0.91-1.47) | 1.00 (0.79-1.27) | 1.06 (0.89-1.25) | 1.35 (1.00-1.82) | 0.931 |
| **X Diseases of the respiratory system** **excluding 2,487 participants with tuberculosis at baseline)** | | | | | | | | |
| Premature death | 336 | **6.02 (3.98-9.09)** | **1.86 (1.23-2.82)** | **1.57 (1.25-1.96)** | 1.00 (0.74-1.34) | 1.04 (0.80-1.36) | 1.11 (0.70-1.77) | 0.001 |
| Death at or after the age of 70 | 1,424 | **2.38 (1.94-2.91)** | 1.25 (1.04-1.51) | 1.10 (0.99-1.23) | 1.00 (0.88-1.14) | 0.86 (0.75-0.98) | 1.00 (0.80-1.26) | <0.001 |
| All death | 1,760 | **2.80 (2.33-3.36)** | **1.35 (1.14-1.60)** | **1.18 (1.07-1.30)** | 1.00 (0.89-1.13) | 0.89 (0.79-1.00) | 1.02 (0.83-1.25) | <0.001 |
| **XI Diseases of the digestive system (excluding 26,069 participants with hepatitis/cirrhosis, peptic ulcer, or gallstones/gallbladder disease at baseline)** | | | | | | | | |
| Premature death | 142 | **5.60 (2.75-11.44)** | 1.88 (0.95-3.74) | 1.33 (0.91-1.95) | 1.00 (0.66-1.51) | 0.87 (0.60-1.26) | 0.70 (0.35-1.37) | 0.006 |
| Death at or after the age of 70 | 293 | 1.16 (0.61-2.20) | 1.32 (0.84-2.07) | 1.10 (0.84-1.44) | 1.00 (0.77-1.31) | 0.84 (0.65-1.08) | 0.75 (0.47-1.18) | 0.118 |
| All death | 435 | **2.04 (1.27-3.29)** | 1.50 (1.03-2.18) | 1.18 (0.95-1.47) | 1.00 (0.80-1.25) | 0.84 (0.69-1.04) | 0.73 (0.50-1.07) | 0.005 |
| **XIV Diseases of the genitourinary system (excluding 3,741 participants with chronic kidney disease at baseline)** | | | | | | | | |
| Premature death | 127 | **2.50 (1.09-5.70)** | 1.86 (0.99-3.48) | 1.01 (0.67-1.52) | 1.00 (0.68-1.48) | 0.68 (0.45-1.02) | 0.76 (0.38-1.55) | 0.062 |
| Death at or after the age of 70 | 170 | 1.46 (0.68-3.14) | 1.39 (0.77-2.50) | 1.08 (0.76-1.55) | 1.00 (0.69-1.44) | 1.01 (0.73-1.41) | 0.87 (0.47-1.60) | 0.397 |
| All death | 297 | 1.85 (1.05-3.26) | 1.58 (1.03-2.44) | 1.05 (0.80-1.38) | 1.00 (0.77-1.31) | 0.86 (0.66-1.11) | 0.83 (0.52-1.31) | 0.069 |

ICD-10, International Classification of Diseases, 10th Revision.

The models were adjusted for the same covariates as described in Figure 1. Linear trend tests were performed with the median value within each body mass index group. Bold indicates *P*<0.05.

**Table S5. Associations between BMI and ICD-10 chapter-specific mortality after excluding those who died within the first five years of follow-up**

| **Chapter** | **No. of deaths** | **<18.5 kg/m^2^** | **18.5-19.9 kg/m^2^** | **20.0-22.4 kg/m^2^** | **22.5-23.9 kg/m^2^** | **24.0-27.9 kg/m^2^** | **≥28.0 kg/m^2^** | ***P*_trend_** |
| --- | --- | --- | --- | --- | --- | --- | --- | --- |
| **I Certain infectious and parasitic diseases** | | | | | | | | |
| Premature death | 113 | 0.43 (0.12-1.52) | 0.38 (0.15-1.00) | 0.69 (0.44-1.08) | 1.00 (0.70-1.43) | 0.89 (0.60-1.31) | 1.05 (0.49-2.23) | 0.202 |
| Death at or after the age of 70 | 91 | **2.93 (1.24-6.91)** | 1.89 (0.92-3.88) | 1.13 (0.70-1.82) | 1.00 (0.58-1.73) | 1.19 (0.73-1.94) | 0.96 (0.39-2.36) | 0.296 |
| All death | 204 | 1.19 (0.60-2.34) | 0.83 (0.47-1.47) | 0.83 (0.60-1.15) | 1.00 (0.74-1.35) | 0.97 (0.72-1.32) | 0.95 (0.53-1.70) | 0.872 |
| **II Neoplasms** | | | | | | | | |
| Premature death | 3,591 | 1.11 (0.91-1.36) | 1.04 (0.91-1.20) | **1.12 (1.04-1.21)** | 1.00 (0.93-1.08) | 1.05 (0.98-1.12) | **1.24 (1.08-1.41)** | 0.360 |
| Death at or after the age of 70 | 3,462 | **1.39 (1.17-1.64)** | 1.01 (0.87-1.16) | 1.03 (0.95-1.12) | 1.00 (0.92-1.08) | 1.00 (0.93-1.07) | 1.03 (0.90-1.17) | 0.377 |
| All death | 7,053 | **1.26 (1.11-1.43)** | 1.03 (0.93-1.14) | 1.08 (1.02-1.14) | 1.00 (0.95-1.06) | 1.02 (0.97-1.08) | **1.13 (1.03-1.23)** | 0.994 |
| **IV Endocrine, nutritional and metabolic diseases** | | | | | | | | |
| Premature death | 379 | **4.33 (2.58-7.25)** | **2.78 (1.88-4.12)** | 1.35 (1.05-1.73) | 1.00 (0.78-1.29) | **0.68 (0.55-0.84)** | **0.56 (0.38-0.81)** | <0.001 |
| Death at or after the age of 70 | 799 | 1.45 (0.97-2.17) | 0.84 (0.58-1.20) | 1.10 (0.92-1.32) | 1.00 (0.85-1.18) | **0.79 (0.69-0.90)** | **0.68 (0.53-0.87)** | 0.005 |
| All death | 1,178 | **2.07 (1.51-2.86)** | 1.30 (1.00-1.70) | 1.18 (1.02-1.36) | 1.00 (0.87-1.15) | **0.75 (0.67-0.84)** | **0.63 (0.52-0.78)** | <0.001 |
| **V Mental and behavioural disorders** | | | | | | | | |
| Premature death | / | / | / | / | / | / | / | / |
| Death at or after the age of 70 | 130 | 1.77 (0.88-3.58) | 1.71 (1.00-2.94) | 1.17 (0.84-1.65) | 1.00 (0.64-1.57) | 1.09 (0.69-1.71) | 0.62 (0.25-1.56) | 0.143 |
| All death | 157 | 1.67 (0.84-3.32) | 1.52 (0.89-2.59) | 1.19 (0.86-1.64) | 1.00 (0.68-1.48) | 1.03 (0.70-1.53) | 0.72 (0.34-1.54) | 0.175 |
| **VI Diseases of the nervous system** | | | | | | | | |
| Premature death | 98 | 1.35 (0.49-3.71) | 1.32 (0.65-2.69) | 1.13 (0.75-1.71) | 1.00 (0.61-1.63) | 1.44 (0.90-2.30) | 0.88 (0.31-2.46) | 0.912 |
| Death at or after the age of 70 | 262 | 1.06 (0.64-1.75) | 1.15 (0.78-1.71) | 0.95 (0.74-1.21) | 1.00 (0.74-1.36) | 1.11 (0.81-1.53) | 1.27 (0.71-2.29) | 0.683 |
| All death | 360 | 1.13 (0.72-1.77) | 1.20 (0.85-1.69) | 1.00 (0.81-1.23) | 1.00 (0.77-1.29) | 1.22 (0.94-1.58) | 1.19 (0.72-1.98) | 0.714 |
| **IX Diseases of the circulatory system** | | | | | | | | |
| Premature death | 2,847 | **1.82 (1.49-2.23)** | 0.96 (0.80-1.15) | 1.08 (0.99-1.19) | 1.00 (0.91-1.09) | 0.99 (0.92-1.06) | **1.18 (1.03-1.35)** | 0.786 |
| Death at or after the age of 70 | 8,699 | **1.30 (1.18-1.44)** | **1.16 (1.07-1.26)** | 1.02 (0.97-1.07) | 1.00 (0.95-1.05) | 1.01 (0.96-1.06) | **1.11 (1.02-1.20)** | 0.434 |
| All death | 11,546 | **1.40 (1.28-1.54)** | **1.13 (1.05-1.22)** | 1.03 (0.99-1.08) | 1.00 (0.96-1.05) | 1.01 (0.97-1.05) | **1.13 (1.06-1.22)** | 0.445 |
| **X Diseases of the respiratory system** | | | | | | | | |
| Premature death | 256 | **6.12 (3.68-10.17)** | 1.84 (1.09-3.09) | **1.74 (1.34-2.27)** | 1.00 (0.72-1.40) | 1.10 (0.83-1.46) | 0.93 (0.55-1.57) | 0.001 |
| Death at or after the age of 70 | 1,345 | **2.46 (1.99-3.03)** | **1.31 (1.08-1.59)** | 1.08 (0.96-1.21) | 1.00 (0.87-1.14) | 0.84 (0.73-0.96) | 1.01 (0.80-1.28) | <0.001 |
| All death | 1,601 | **2.80 (2.31-3.41)** | **1.39 (1.16-1.67)** | 1.17 (1.05-1.30) | 1.00 (0.88-1.13) | 0.88 (0.78-1.00) | 1.00 (0.81-1.24) | <0.001 |
| **XI Diseases of the digestive system** | | | | | | | | |
| Premature death | 144 | **5.62 (2.65-11.93)** | 1.70 (0.80-3.60) | **1.83 (1.28-2.62)** | 1.00 (0.66-1.52) | 0.92 (0.64-1.32) | 0.63 (0.32-1.27) | 0.002 |
| Death at or after the age of 70 | 345 | 1.55 (0.90-2.68) | 1.49 (0.99-2.24) | 1.32 (1.05-1.67) | 1.00 (0.77-1.30) | 0.91 (0.71-1.16) | 0.86 (0.56-1.32) | 0.058 |
| All death | 489 | **2.25 (1.44-3.50)** | **1.59 (1.11-2.27)** | **1.46 (1.20-1.78)** | 1.00 (0.80-1.25) | 0.91 (0.75-1.12) | 0.80 (0.55-1.15) | 0.001 |
| **XIII Diseases of the musculoskeletal system and connective tissue** | | | | | | | | |
| Premature death | / | / | / | / | / | / | / | / |
| Death at or after the age of 70 | / | / | / | / | / | / | / | / |
| All death | 103 | 1.32 (0.54-3.20) | 1.15 (0.57-2.31) | 0.84 (0.54-1.30) | 1.00 (0.65-1.54) | 0.75 (0.47-1.22) | 1.51 (0.69-3.29) | 0.788 |
| **XIV Diseases of the genitourinary system** | | | | | | | | |
| Premature death | 100 | 2.21 (0.85-5.73) | 1.16 (0.51-2.66) | 1.01 (0.63-1.61) | 1.00 (0.64-1.55) | 0.73 (0.47-1.14) | 1.01 (0.47-2.16) | 0.475 |
| Death at or after the age of 70 | 165 | 1.97 (0.93-4.16) | 1.51 (0.82-2.76) | 1.12 (0.77-1.61) | 1.00 (0.69-1.45) | 0.99 (0.71-1.39) | 0.79 (0.42-1.47) | 0.183 |
| All death | 265 | **2.03 (1.13-3.67)** | 1.37 (0.84-2.23) | 1.07 (0.80-1.43) | 1.00 (0.75-1.33) | 0.89 (0.68-1.17) | 0.89 (0.55-1.43) | 0.155 |
| **XVIII Symptoms, signs and abnormal clinical and laboratory findings, not elsewhere classified** | | | | | | | | |
| Premature death | 165 | 0.95 (0.32-2.76) | 1.15 (0.59-2.24) | 1.06 (0.73-1.53) | 1.00 (0.72-1.40) | 0.84 (0.61-1.15) | 0.77 (0.42-1.41) | 0.383 |
| Death at or after the age of 70 | 392 | 1.25 (0.77-2.06) | 1.25 (0.86-1.82) | 0.94 (0.74-1.19) | 1.00 (0.80-1.24) | 0.75 (0.60-0.94) | 0.74 (0.50-1.11) | 0.079 |
| All death | 557 | 1.22 (0.78-1.89) | 1.23 (0.88-1.70) | 0.97 (0.80-1.18) | 1.00 (0.83-1.20) | 0.78 (0.65-0.94) | 0.76 (0.54-1.06) | 0.056 |
| **XX External causes of morbidity and mortality** | | | | | | | | |
| Premature death | 713 | 1.50 (1.03-2.18) | 1.20 (0.91-1.58) | 1.01 (0.86-1.19) | 1.00 (0.85-1.18) | 0.91 (0.77-1.08) | 0.81 (0.58-1.13) | 0.064 |
| Death at or after the age of 70 | 784 | 1.38 (1.02-1.87) | 1.30 (1.03-1.65) | 0.99 (0.86-1.15) | 1.00 (0.84-1.18) | 0.87 (0.72-1.04) | 0.98 (0.72-1.34) | 0.116 |
| All death | 1,497 | **1.42 (1.12-1.80)** | **1.25 (1.05-1.50)** | 1.00 (0.90-1.12) | 1.00 (0.89-1.12) | 0.89 (0.78-1.01) | 0.89 (0.71-1.12) | 0.015 |

ICD-10, International Classification of Diseases, 10th Revision.

The models were adjusted for the same covariates as described in Figure 1. Linear trend tests were performed with the median value within each body mass index group. Bold indicates *P*<0.05.

**Table S6. Associations between BMI and cause-specific mortality**

| **Disease** | **No. of deaths** | **<18.5 kg/m^2^** | **18.5-19.9 kg/m^2^** | **20.0-22.4 kg/m^2^** | **22.5-23.9 kg/m^2^** | **24.0-27.9 kg/m^2^** | **≥28.0 kg/m^2^** | ***P*_trend_** |
| --- | --- | --- | --- | --- | --- | --- | --- | --- |
| **Viral hepatitis** | | | | | | | | |
| Premature death | 93 | 0.46 (0.10-2.06) | 0.35 (0.11-1.17) | 1.04 (0.65-1.67) | 1.00 (0.65-1.55) | 0.99 (0.65-1.51) | 1.04 (0.46-2.37) | 0.443 |
| Death at or after the age of 70 | / | / | / | / | / | / | / | / |
| All death | 140 | 0.74 (0.29-1.89) | 0.46 (0.19-1.09) | 0.89 (0.60-1.33) | 1.00 (0.69-1.45) | 1.18 (0.84-1.65) | 1.27 (0.66-2.43) | 0.170 |
| **Less-common certain infectious and parasitic diseases combined^*^** | | | | | | | | |
| Premature death | / | / | / | / | / | / | / | / |
| Death at or after the age of 70 | / | / | / | / | / | / | / | / |
| All death | 127 | **3.26 (1.63-6.54)^†^** | 2.02 (1.15-3.56) | 1.22 (0.86-1.72) | 1.00 (0.65-1.53) | 0.69 (0.43-1.10) | 0.65 (0.29-1.49) | 0.008 |
| **Malignant neoplasms of lip, oral cavity and pharynx** | | | | | | | | |
| Premature death | / | / | / | / | / | / | / | / |
| Death at or after the age of 70 | / | / | / | / | / | / | / | / |
| All death | 94 | 1.93 (0.77-4.81) | 0.64 (0.22-1.81) | 1.31 (0.84-2.05) | 1.00 (0.58-1.72) | 1.81 (1.16-2.82) | 1.25 (0.49-3.16) | 0.637 |
| **Malignant neoplasm of oesophagus** | | | | | | | | |
| Premature death | 235 | **2.29 (1.13-4.65)** | 1.70 (1.01-2.88) | 1.36 (1.00-1.85) | 1.00 (0.72-1.39) | 1.06 (0.82-1.38) | 0.83 (0.51-1.35) | 0.054 |
| Death at or after the age of 70 | 267 | **1.86 (1.14-3.03)** | 1.34 (0.89-2.00) | 0.98 (0.76-1.27) | 1.00 (0.75-1.33) | 0.92 (0.68-1.23) | 0.72 (0.42-1.24) | 0.059 |
| All death | 502 | **2.13 (1.43-3.18)^†^** | **1.53 (1.11-2.10)** | 1.14 (0.94-1.39) | 1.00 (0.81-1.24) | 0.99 (0.81-1.21) | 0.79 (0.55-1.14) | 0.007 |
| **Malignant neoplasm of stomach** | | | | | | | | |
| Premature death | 448 | 1.22 (0.73-2.05) | 0.94 (0.62-1.41) | 1.15 (0.93-1.42) | 1.00 (0.80-1.24) | 1.08 (0.88-1.32) | 1.13 (0.77-1.66) | 0.927 |
| Death at or after the age of 70 | 396 | **2.40 (1.56-3.71)^†^** | **1.81 (1.26-2.59)** | 1.24 (0.98-1.57) | 1.00 (0.76-1.32) | 1.26 (1.00-1.58) | 1.45 (0.98-2.14) | 0.481 |
| All death | 844 | **1.73 (1.25-2.41)^†^** | 1.30 (0.99-1.70) | 1.19 (1.02-1.39) | 1.00 (0.84-1.19) | 1.15 (0.99-1.34) | 1.27 (0.97-1.67) | 0.670 |
| **Malignant neoplasm of colon and rectum** | | | | | | | | |
| Premature death | 394 | 1.80 (1.02-3.15) | 1.05 (0.66-1.68) | **1.44 (1.15-1.81)** | 1.00 (0.78-1.28) | 1.09 (0.89-1.35) | 1.10 (0.74-1.64) | 0.353 |
| Death at or after the age of 70 | 418 | 1.27 (0.76-2.11) | 0.79 (0.50-1.27) | 0.99 (0.77-1.27) | 1.00 (0.79-1.27) | 1.11 (0.92-1.35) | 1.27 (0.90-1.81) | 0.321 |
| All death | 812 | 1.49 (1.03-2.18) | 0.91 (0.65-1.26) | 1.20 (1.02-1.42) | 1.00 (0.84-1.19) | 1.11 (0.96-1.28) | 1.21 (0.93-1.57) | 0.884 |
| **Malignant neoplasm of liver and intrahepatic bile ducts** | | | | | | | | |
| Premature death | 590 | 1.49 (0.98-2.26) | 1.02 (0.73-1.44) | 1.09 (0.91-1.31) | 1.00 (0.83-1.21) | 1.04 (0.87-1.25) | 1.22 (0.88-1.70) | 0.980 |
| Death at or after the age of 70 | 441 | 1.37 (0.86-2.18) | 1.34 (0.93-1.92) | 1.03 (0.82-1.29) | 1.00 (0.79-1.27) | 1.21 (0.98-1.48) | 1.39 (0.97-1.99) | 0.566 |
| All death | 1,031 | **1.41 (1.04-1.93)** | 1.16 (0.91-1.49) | 1.07 (0.93-1.24) | 1.00 (0.86-1.16) | 1.10 (0.97-1.26) | 1.28 (1.00-1.63) | 0.714 |
| **Malignant neoplasm of gallbladder and biliary tract** | | | | | | | | |
| Premature death | 123 | 1.34 (0.52-3.43) | 1.12 (0.55-2.28) | 1.01 (0.67-1.51) | 1.00 (0.67-1.49) | 0.91 (0.61-1.36) | 0.95 (0.45-2.01) | 0.670 |
| Death at or after the age of 70 | 139 | 0.97 (0.44-2.15) | 0.76 (0.39-1.51) | 0.75 (0.50-1.13) | 1.00 (0.68-1.46) | 0.94 (0.64-1.37) | 1.34 (0.70-2.56) | 0.333 |
| All death | 262 | 1.12 (0.61-2.06) | 0.92 (0.56-1.51) | 0.87 (0.65-1.16) | 1.00 (0.76-1.32) | 0.92 (0.70-1.21) | 1.14 (0.70-1.86) | 0.710 |
| **Malignant neoplasm of pancreas** | | | | | | | | |
| Premature death | 241 | 1.49 (0.72-3.06) | 0.95 (0.52-1.74) | 1.26 (0.93-1.71) | 1.00 (0.72-1.39) | 1.45 (1.12-1.87) | 1.72 (1.05-2.81) | 0.267 |
| Death at or after the age of 70 | 268 | 0.90 (0.43-1.92) | 0.96 (0.56-1.66) | 0.72 (0.52-1.01) | 1.00 (0.77-1.30) | 0.83 (0.66-1.05) | 0.72 (0.46-1.12) | 0.623 |
| All death | 509 | 1.13 (0.67-1.91) | 0.96 (0.64-1.43) | 0.95 (0.76-1.19) | 1.00 (0.81-1.23) | 1.05 (0.89-1.25) | 1.05 (0.75-1.46) | 0.743 |
| **Malignant neoplasm of trachea, bronchus and lung** | | | | | | | | |
| Premature death | 981 | 1.24 (0.86-1.80) | 1.19 (0.91-1.54) | **1.32 (1.15-1.52)** | 1.00 (0.86-1.16) | 1.06 (0.92-1.21) | 1.03 (0.79-1.34) | 0.196 |
| Death at or after the age of 70 | 840 | 1.18 (0.83-1.67) | 1.03 (0.78-1.37) | 1.14 (0.98-1.33) | 1.00 (0.85-1.17) | 0.88 (0.76-1.03) | 0.83 (0.63-1.09) | 0.086 |
| All death | 1,821 | 1.22 (0.94-1.57) | 1.12 (0.92-1.35) | **1.24 (1.12-1.38)** | 1.00 (0.90-1.12) | 0.97 (0.88-1.08) | 0.93 (0.77-1.12) | 0.031 |
| **Malignant neoplasm of breast** | | | | | | | | |
| Premature death | 406 | 1.42 (0.80-2.54) | 0.80 (0.49-1.31) | 1.05 (0.82-1.34) | 1.00 (0.79-1.27) | 1.14 (0.94-1.38) | 1.49 (1.04-2.12) | 0.179 |
| Death at or after the age of 70 | 137 | 2.07 (0.78-5.51) | 1.00 (0.38-2.62) | 1.32 (0.81-2.15) | 1.00 (0.60-1.66) | 1.47 (1.12-1.92) | 1.75 (1.03-2.95) | 0.441 |
| All death | 543 | 1.55 (0.94-2.55) | 0.84 (0.54-1.31) | 1.11 (0.89-1.38) | 1.00 (0.81-1.24) | 1.20 (1.02-1.40) | **1.52 (1.13-2.05)** | 0.135 |
| **Malignant neoplasm of cervix uteri** | | | | | | | | |
| Premature death | 225 | 1.50 (0.72-3.10) | 1.66 (1.04-2.67) | **1.53 (1.18-1.97)** | 1.00 (0.73-1.36) | 0.85 (0.61-1.17) | 0.61 (0.33-1.15) | 0.009 |
| Death at or after the age of 70 | 96 | 1.37 (0.56-3.35) | 0.91 (0.40-2.04) | 1.61 (1.12-2.32) | 1.00 (0.58-1.71) | 1.10 (0.66-1.85) | 0.98 (0.37-2.57) | 0.637 |
| All death | 321 | 1.50 (0.86-2.62) | 1.40 (0.93-2.11) | **1.56 (1.27-1.93)** | 1.00 (0.76-1.31) | 0.92 (0.70-1.21) | 0.71 (0.42-1.20) | 0.014 |
| **Malignant neoplasm of corpus uteri** | | | | | | | | |
| Premature death | 83 | 1.62 (0.57-4.61) | 0.99 (0.40-2.40) | 1.17 (0.74-1.86) | 1.00 (0.61-1.65) | 0.90 (0.55-1.49) | 1.26 (0.51-3.08) | 0.848 |
| Death at or after the age of 70 | / | / | / | / | / | / | / | / |
| All death | 114 | 1.47 (0.59-3.67) | 0.86 (0.38-1.95) | 1.03 (0.67-1.59) | 1.00 (0.64-1.55) | 1.16 (0.78-1.72) | 1.37 (0.66-2.84) | 0.633 |
| **Malignant neoplasm of uterus, part unspecified** | | | | | | | | |
| Premature death | 95 | 1.17 (0.34-4.08) | 2.09 (1.03-4.23) | 1.36 (0.87-2.13) | 1.00 (0.59-1.69) | 0.91 (0.56-1.46) | 1.87 (0.87-4.01) | 0.969 |
| Death at or after the age of 70 | / | / | / | / | / | / | / | / |
| All death | 128 | 2.48 (1.11-5.54) | **2.34 (1.29-4.24)** | 1.46 (1.00-2.13) | 1.00 (0.61-1.64) | 1.14 (0.75-1.74) | 1.92 (0.97-3.80) | 0.735 |
| **Malignant neoplasm of ovary** | | | | | | | | |
| Premature death | 169 | 1.17 (0.44-3.10) | 1.01 (0.51-2.01) | 1.25 (0.88-1.76) | 1.00 (0.70-1.42) | 1.09 (0.80-1.50) | 0.80 (0.42-1.53) | 0.503 |
| Death at or after the age of 70 | 80 | 1.65 (0.50-5.48) | 0.93 (0.31-2.77) | 1.13 (0.66-1.96) | 1.00 (0.59-1.68) | 0.90 (0.57-1.40) | 0.84 (0.37-1.92) | 0.553 |
| All death | 249 | 1.35 (0.64-2.86) | 0.99 (0.55-1.76) | 1.21 (0.90-1.62) | 1.00 (0.75-1.34) | 1.03 (0.79-1.33) | 0.83 (0.50-1.37) | 0.369 |
| **Malignant neoplasms of central nervous system** | | | | | | | | |
| Premature death | 143 | 0.34 (0.10-1.17) | **0.31 (0.12-0.80)** | 0.85 (0.57-1.26) | 1.00 (0.70-1.43) | 1.22 (0.88-1.69) | 1.98 (1.06-3.71) | 0.005 |
| Death at or after the age of 70 | 80 | 1.10 (0.40-3.01) | 1.02 (0.46-2.25) | 0.48 (0.26-0.89) | 1.00 (0.62-1.61) | 0.96 (0.58-1.57) | 0.79 (0.31-2.01) | 0.952 |
| All death | 223 | 0.63 (0.30-1.31) | 0.56 (0.31-1.00) | 0.72 (0.52-0.99) | 1.00 (0.75-1.33) | 1.14 (0.86-1.51) | 1.46 (0.86-2.48) | 0.021 |
| **Malignant neoplasms, stated or presumed to be primary, of lymphoid, haematopoietic and related tissue** | | | | | | | | |
| Premature death | 276 | 0.57 (0.25-1.29) | 1.16 (0.75-1.81) | 0.82 (0.62-1.08) | 1.00 (0.78-1.29) | 0.91 (0.70-1.19) | 1.41 (0.88-2.26) | 0.259 |
| Death at or after the age of 70 | 215 | **0.28 (0.10-0.82)** | **0.47 (0.24-0.93)** | 0.88 (0.64-1.23) | 1.00 (0.75-1.33) | 0.83 (0.64-1.09) | 1.07 (0.65-1.77) | 0.191 |
| All death | 491 | **0.42 (0.22-0.81)^†^** | 0.85 (0.59-1.23) | 0.85 (0.69-1.05) | 1.00 (0.83-1.21) | 0.87 (0.72-1.05) | 1.22 (0.87-1.73) | 0.100 |
| **Less-common neoplasms combined^*^** | | | | | | | | |
| Premature death | 363 | **2.05 (1.21-3.48)^†^** | 1.44 (0.95-2.18) | 0.88 (0.67-1.15) | 1.00 (0.78-1.28) | 1.14 (0.92-1.42) | 0.99 (0.65-1.50) | 0.489 |
| Death at or after the age of 70 | 444 | **2.63 (1.72-4.03)^†^** | 0.80 (0.50-1.29) | 1.21 (0.97-1.52) | 1.00 (0.79-1.26) | 0.98 (0.80-1.20) | 0.92 (0.64-1.33) | 0.106 |
| All death | 743 | **2.28 (1.61-3.23)^†^** | 1.06 (0.76-1.46) | 1.05 (0.88-1.26) | 1.00 (0.84-1.19) | 0.99 (0.85-1.16) | 0.90 (0.68-1.20) | 0.073 |
| **Type 2 diabetes mellitus** | | | | | | | | |
| Premature death | 443 | **5.72 (3.80-8.60)^†^** | **2.89 (2.06-4.06)^†^** | 1.27 (1.01-1.58) | 1.00 (0.80-1.25) | **0.61 (0.50-0.76)** | **0.41 (0.28-0.60)^†^** | <0.001 |
| Death at or after the age of 70 | 745 | **1.62 (1.08-2.43)** | 0.80 (0.54-1.18) | 1.19 (0.99-1.42) | 1.00 (0.84-1.19) | 0.82 (0.71-0.94) | **0.66 (0.51-0.86)** | 0.002 |
| All death | 1,188 | **2.69 (2.01-3.59)^†^** | **1.43 (1.11-1.84)** | **1.22 (1.06-1.41)** | 1.00 (0.87-1.15) | **0.73 (0.65-0.83)** | **0.55 (0.45-0.68)** | <0.001 |
| **Less-common endocrine, nutritional and metabolic diseases combined^*^** | | | | | | | | |
| Premature death | / | / | / | / | / | / | / | / |
| Death at or after the age of 70 | 119 | 1.18 (0.44-3.17) | 1.25 (0.60-2.60) | 1.06 (0.68-1.65) | 1.00 (0.66-1.52) | 0.77 (0.52-1.14) | 0.86 (0.44-1.71) | 0.475 |
| All death | 168 | **2.40 (1.16-4.97)** | **1.98 (1.14-3.44)** | 0.97 (0.66-1.43) | 1.00 (0.70-1.43) | 0.72 (0.51-1.02) | 0.88 (0.49-1.56) | 0.106 |
| **Other degenerative diseases of nervous system, not elsewhere classified** | | | | | | | | |
| Premature death | / | / | / | / | / | / | / | / |
| Death at or after the age of 70 | 114 | 0.96 (0.47-1.96) | 0.92 (0.51-1.68) | 1.15 (0.84-1.59) | 1.00 (0.61-1.65) | 1.11 (0.66-1.87) | 2.10 (0.86-5.16) | 0.360 |
| All death | 134 | 1.04 (0.53-2.04) | 0.92 (0.53-1.61) | 1.01 (0.74-1.39) | 1.00 (0.65-1.54) | 1.03 (0.65-1.65) | 1.62 (0.71-3.72) | 0.537 |
| **Less-common diseases of the nervous system combined^*^** | | | | | | | | |
| Premature death | / | / | / | / | / | / | / | / |
| Death at or after the age of 70 | 161 | 1.09 (0.56-2.12) | 1.28 (0.77-2.12) | 0.85 (0.61-1.18) | 1.00 (0.70-1.43) | 0.93 (0.63-1.38) | 0.73 (0.33-1.58) | 0.491 |
| All death | 251 | 1.26 (0.72-2.19) | 1.36 (0.90-2.06) | 1.05 (0.82-1.36) | 1.00 (0.74-1.36) | 1.19 (0.88-1.63) | 0.84 (0.44-1.58) | 0.557 |
| **Chronic rheumatic heart diseases** | | | | | | | | |
| Premature death | / | / | / | / | / | / | / | / |
| Death at or after the age of 70 | / | / | / | / | / | / | / | / |
| All death | 109 | **2.38 (1.16-4.85)** | 0.65 (0.29-1.46) | 0.92 (0.61-1.38) | 1.00 (0.63-1.59) | 0.94 (0.58-1.51) | 1.60 (0.72-3.56) | 0.919 |
| **Hypertensive diseases** | | | | | | | | |
| Premature death | 115 | 1.02 (0.30-3.54) | 1.27 (0.58-2.78) | 0.99 (0.63-1.56) | 1.00 (0.67-1.49) | 0.59 (0.39-0.87) | 1.02 (0.53-1.94) | 0.665 |
| Death at or after the age of 70 | 767 | 1.01 (0.72-1.42) | 0.87 (0.65-1.16) | 0.91 (0.77-1.07) | 1.00 (0.85-1.18) | 1.10 (0.94-1.29) | 1.19 (0.88-1.59) | 0.170 |
| All death | 882 | 1.04 (0.75-1.44) | 0.91 (0.70-1.20) | 0.92 (0.79-1.07) | 1.00 (0.86-1.17) | 1.02 (0.88-1.18) | 1.20 (0.92-1.57) | 0.242 |
| **Ischaemic heart diseases other than myocardial infarction** | | | | | | | | |
| Premature death | 361 | **3.25 (1.91-5.51)^†^** | 1.48 (0.91-2.40) | 1.31 (1.00-1.71) | 1.00 (0.76-1.32) | 0.89 (0.72-1.10) | 1.41 (0.99-2.01) | 0.512 |
| Death at or after the age of 70 | 1,655 | **1.70 (1.33-2.17)^†^** | 1.21 (0.98-1.50) | 1.16 (1.02-1.31) | 1.00 (0.88-1.14) | 1.05 (0.95-1.16) | 1.09 (0.92-1.30) | 0.226 |
| All death | 2,016 | **1.91 (1.53-2.38)^†^** | **1.27 (1.04-1.54)** | **1.18 (1.06-1.33)** | 1.00 (0.89-1.12) | 1.02 (0.94-1.12) | 1.15 (0.99-1.35) | 0.171 |
| **Myocardial infarction** | | | | | | | | |
| Premature death | 889 | **3.29 (2.36-4.60)^†^** | **1.83 (1.38-2.43)^†^** | **1.52 (1.30-1.79)^†^** | 1.00 (0.83-1.20) | 1.24 (1.09-1.42) | 1.12 (0.88-1.43) | 0.003 |
| Death at or after the age of 70 | 2,037 | **1.50 (1.23-1.84)^†^** | 1.18 (1.00-1.40) | 1.06 (0.96-1.17) | 1.00 (0.90-1.11) | **0.86 (0.77-0.95)** | 0.99 (0.83-1.17) | 0.022 |
| All death | 2,926 | **1.86 (1.57-2.21)^†^** | **1.35 (1.17-1.56)** | **1.18 (1.08-1.28)** | 1.00 (0.91-1.10) | 0.96 (0.88-1.04) | 1.02 (0.88-1.17) | <0.001 |
| **Cardiac arrest** | | | | | | | | |
| Premature death | / | / | / | / | / | / | / | / |
| Death at or after the age of 70 | / | / | / | / | / | / | / | / |
| All death | 97 | 1.69 (0.65-4.39) | 0.80 (0.31-2.08) | 1.22 (0.78-1.90) | 1.00 (0.63-1.60) | 0.76 (0.48-1.21) | 1.69 (0.82-3.48) | 0.746 |
| **Subarachnoid haemorrhage** | | | | | | | | |
| Premature death | / | / | / | / | / | / | / | / |
| Death at or after the age of 70 | / | / | / | / | / | / | / | / |
| All death | 125 | 1.27 (0.41-3.90) | 1.14 (0.50-2.61) | 1.19 (0.76-1.86) | 1.00 (0.64-1.56) | 1.34 (0.96-1.87) | 0.91 (0.46-1.83) | 0.812 |
| **Intracerebral haemorrhage** | | | | | | | | |
| Premature death | 1,375 | **1.52 (1.15-2.01)^†^** | 0.87 (0.68-1.11) | 0.96 (0.84-1.10) | 1.00 (0.88-1.13) | 1.05 (0.94-1.17) | **1.32 (1.08-1.61)** | 0.165 |
| Death at or after the age of 70 | 1,780 | **1.33 (1.09-1.62)** | **1.26 (1.07-1.47)** | 1.03 (0.94-1.14) | 1.00 (0.89-1.12) | 1.04 (0.93-1.17) | 0.93 (0.75-1.16) | 0.072 |
| All death | 3,155 | **1.39 (1.19-1.64)^†^** | 1.12 (0.99-1.28) | 1.00 (0.93-1.09) | 1.00 (0.92-1.09) | 1.05 (0.97-1.14) | 1.13 (0.98-1.31) | 0.697 |
| **Cerebral infarction** | | | | | | | | |
| Premature death | 332 | 0.96 (0.45-2.03) | 1.21 (0.75-1.96) | 1.29 (1.00-1.68) | 1.00 (0.77-1.30) | 0.86 (0.69-1.08) | 1.11 (0.75-1.65) | 0.595 |
| Death at or after the age of 70 | 1,164 | 1.14 (0.86-1.50) | 1.15 (0.93-1.43) | 0.95 (0.83-1.09) | 1.00 (0.87-1.15) | 1.01 (0.88-1.15) | 1.22 (0.98-1.53) | 0.503 |
| All death | 1,496 | 1.14 (0.88-1.48) | 1.17 (0.96-1.43) | 1.02 (0.90-1.15) | 1.00 (0.88-1.13) | 0.97 (0.87-1.09) | 1.21 (0.99-1.47) | 0.714 |
| **Stroke, not specified as haemorrhage or infarction** | | | | | | | | |
| Premature death | / | / | / | / | / | / | / | / |
| Death at or after the age of 70 | 114 | 0.55 (0.20-1.50) | 1.04 (0.54-2.02) | 1.23 (0.85-1.79) | 1.00 (0.64-1.57) | 1.15 (0.74-1.79) | 1.28 (0.57-2.88) | 0.575 |
| All death | 165 | 0.59 (0.25-1.40) | 0.76 (0.42-1.40) | 0.82 (0.59-1.15) | 1.00 (0.74-1.35) | **0.63 (0.44-0.91)** | 0.65 (0.33-1.26) | 0.596 |
| **Other cerebrovascular diseases** | | | | | | | | |
| Premature death | / | / | / | / | / | / | / | / |
| Death at or after the age of 70 | / | / | / | / | / | / | / | / |
| All death | 101 | 1.44 (0.46-4.53) | 1.82 (0.84-3.95) | 1.13 (0.68-1.86) | 1.00 (0.62-1.62) | 1.15 (0.78-1.71) | 0.67 (0.30-1.49) | 0.268 |
| **Sequelae of cerebrovascular disease** | | | | | | | | |
| Premature death | 353 | 0.91 (0.41-2.03) | 0.73 (0.40-1.31) | 0.96 (0.72-1.28) | 1.00 (0.78-1.29) | 0.86 (0.72-1.04) | 0.92 (0.65-1.30) | 0.997 |
| Death at or after the age of 70 | 1,339 | 0.81 (0.59-1.13) | 1.20 (0.96-1.50) | 1.01 (0.88-1.16) | 1.00 (0.87-1.15) | **1.20 (1.08-1.34)** | **1.43 (1.18-1.73)** | 0.013 |
| All death | 1,692 | 0.83 (0.62-1.13) | 1.12 (0.91-1.38) | 1.00 (0.88-1.13) | 1.00 (0.89-1.13) | 1.12 (1.02-1.23) | **1.31 (1.11-1.55)** | 0.024 |
| **Less-common diseases of the circulatory system combined^*^** | | | | | | | | |
| Premature death | 294 | 1.09 (0.54-2.20) | 0.76 (0.44-1.33) | 0.92 (0.70-1.22) | 1.00 (0.79-1.27) | 0.80 (0.63-1.02) | 0.81 (0.52-1.27) | 0.599 |
| Death at or after the age of 70 | 460 | 1.56 (1.01-2.39) | 0.93 (0.63-1.38) | 0.92 (0.73-1.15) | 1.00 (0.81-1.24) | 0.90 (0.74-1.11) | 1.06 (0.75-1.51) | 0.839 |
| All death | 380 | 1.50 (0.94-2.39) | 0.78 (0.50-1.22) | 0.88 (0.69-1.12) | 1.00 (0.80-1.26) | 0.86 (0.69-1.08) | 1.16 (0.79-1.72) | 0.764 |
| **Pneumonia** | | | | | | | | |
| Premature death | 86 | 3.13 (1.07-9.21) | 1.54 (0.62-3.81) | 0.80 (0.44-1.45) | 1.00 (0.62-1.63) | 0.85 (0.56-1.30) | 0.74 (0.33-1.64) | 0.284 |
| Death at or after the age of 70 | 395 | **2.79 (1.74-4.45)^†^** | 1.50 (0.99-2.28) | 1.20 (0.94-1.53) | 1.00 (0.79-1.27) | **0.72 (0.58-0.90)** | 0.73 (0.50-1.06) | 0.001 |
| All death | 481 | **2.78 (1.80-4.27)^†^** | 1.50 (1.03-2.20) | 1.12 (0.90-1.40) | 1.00 (0.81-1.24) | **0.75 (0.62-0.91)** | 0.73 (0.52-1.02) | 0.001 |
| **Chronic obstructive pulmonary disease** | | | | | | | | |
| Premature death | 198 | **6.73 (4.12-11.00)^†^** | 1.89 (1.13-3.17) | **1.79 (1.37-2.33)** | 1.00 (0.66-1.51) | 1.11 (0.76-1.62) | 1.31 (0.68-2.50) | 0.004 |
| Death at or after the age of 70 | 868 | **2.18 (1.72-2.75)^†^** | 1.15 (0.93-1.43) | 1.03 (0.90-1.17) | 1.00 (0.84-1.19) | 0.86 (0.71-1.04) | 1.06 (0.76-1.48) | 0.005 |
| All death | 1,066 | **2.65 (2.14-3.28)^†^** | 1.27 (1.04-1.54) | 1.14 (1.01-1.28) | 1.00 (0.85-1.18) | 0.89 (0.75-1.06) | 1.10 (0.82-1.47) | <0.001 |
| **Other respiratory disorders** | | | | | | | | |
| Premature death | / | / | / | / | / | / | / | / |
| Death at or after the age of 70 | 92 | 2.03 (0.68-6.07) | 1.67 (0.70-3.96) | 1.24 (0.73-2.11) | 1.00 (0.56-1.78) | 1.26 (0.85-1.88) | 1.46 (0.72-2.96) | 0.992 |
| All death | 112 | **2.92 (1.18-7.20)** | 1.42 (0.62-3.27) | 1.37 (0.86-2.18) | 1.00 (0.59-1.70) | 1.31 (0.91-1.90) | 1.83 (0.96-3.48) | 0.822 |
| **Less-common diseases of the respiratory system combined^*^** | | | | | | | | |
| Premature death | / | / | / | / | / | / | / | / |
| Death at or after the age of 70 | 109 | 1.52 (0.63-3.67) | 0.86 (0.38-1.97) | 1.00 (0.63-1.59) | 1.00 (0.63-1.60) | 1.13 (0.75-1.69) | 1.08 (0.52-2.26) | 0.946 |
| All death | 147 | 2.29 (1.10-4.80) | 1.46 (0.77-2.76) | 1.37 (0.96-1.97) | 1.00 (0.65-1.53) | 1.07 (0.74-1.55) | 0.88 (0.45-1.74) | 0.186 |
| **Diseases of liver** | | | | | | | | |
| Premature death | 116 | 2.60 (0.97-7.01) | 2.08 (1.03-4.18) | 1.71 (1.18-2.48) | 1.00 (0.65-1.54) | 0.66 (0.42-1.03) | 0.53 (0.24-1.18) | 0.004 |
| Death at or after the age of 70 | 115 | 2.90 (1.12-7.53) | **3.23 (1.69-6.15)** | 1.34 (0.85-2.11) | 1.00 (0.61-1.63) | 0.92 (0.61-1.37) | 0.86 (0.43-1.73) | 0.051 |
| All death | 231 | **2.71 (1.37-5.38)^†^** | **2.62 (1.64-4.19)** | **1.56 (1.17-2.07)** | 1.00 (0.72-1.38) | 0.78 (0.58-1.05) | 0.69 (0.41-1.17) | 0.001 |
| **Less-common diseases of the digestive system combined^*^** | | | | | | | | |
| Premature death | 82 | **11.03 (5.04-24.14)^†^** | 1.78 (0.70-4.56) | 1.65 (1.06-2.58) | 1.00 (0.58-1.73) | 0.74 (0.43-1.28) | 0.40 (0.14-1.12) | <0.001 |
| Death at or after the age of 70 | 253 | 1.14 (0.60-2.15) | 1.00 (0.60-1.67) | 1.27 (0.97-1.66) | 1.00 (0.75-1.34) | 0.90 (0.67-1.19) | 0.80 (0.48-1.35) | 0.266 |
| All death | 335 | **2.22 (1.36-3.63)^†^** | 1.18 (0.75-1.84) | 1.37 (1.09-1.72) | 1.00 (0.77-1.30) | 0.85 (0.66-1.10) | 0.68 (0.42-1.08) | 0.006 |
| **Chronic nephritic syndrome** | | | | | | | | |
| Premature death | / | / | / | / | / | / | / | / |
| Death at or after the age of 70 | 90 | 1.16 (0.43-3.15) | 1.13 (0.53-2.42) | 0.95 (0.60-1.49) | 1.00 (0.64-1.55) | 0.64 (0.38-1.06) | 0.56 (0.23-1.38) | 0.223 |
| All death | 162 | 1.89 (0.96-3.74) | **1.88 (1.14-3.09)** | 1.08 (0.79-1.50) | 1.00 (0.70-1.43) | 0.62 (0.41-0.94) | 0.67 (0.33-1.34) | 0.018 |
| **Less-common diseases of the genitourinary system combined^*^** | | | | | | | | |
| Premature death | / | / | / | / | / | / | / | / |
| Death at or after the age of 70 | 93 | 3.15 (1.14-8.69) | 2.21 (0.94-5.17) | 1.73 (1.05-2.85) | 1.00 (0.53-1.89) | 1.71 (1.13-2.59) | 1.40 (0.64-3.07) | 0.551 |
| All death | 159 | 2.29 (0.99-5.30) | 1.31 (0.63-2.71) | 1.25 (0.85-1.85) | 1.00 (0.67-1.48) | 1.06 (0.78-1.44) | 0.88 (0.49-1.58) | 0.254 |
| **Senility** | | | | | | | | |
| Premature death | / | / | / | / | / | / | / | / |
| Death at or after the age of 70 | 111 | 1.16 (0.49-2.72) | 1.94 (1.09-3.44) | 1.19 (0.82-1.74) | 1.00 (0.63-1.58) | 0.82 (0.50-1.34) | 0.67 (0.28-1.61) | 0.131 |
| All death | 114 | 1.10 (0.47-2.59) | 2.01 (1.14-3.54) | 1.18 (0.81-1.72) | 1.00 (0.63-1.58) | 0.87 (0.53-1.40) | 0.78 (0.33-1.81) | 0.199 |
| **Ill-defined and unknown causes of mortality** | | | | | | | | |
| Premature death | 190 | 2.15 (1.03-4.48) | 1.33 (0.73-2.40) | 1.02 (0.73-1.44) | 1.00 (0.73-1.37) | 0.79 (0.58-1.08) | 0.77 (0.43-1.37) | 0.119 |
| Death at or after the age of 70 | 288 | 1.42 (0.80-2.51) | 0.99 (0.62-1.60) | 0.85 (0.64-1.12) | 1.00 (0.78-1.28) | **0.65 (0.50-0.84)** | 0.74 (0.47-1.16) | 0.159 |
| All death | 478 | **1.67 (1.07-2.62)** | 1.11 (0.77-1.60) | 0.91 (0.74-1.13) | 1.00 (0.83-1.21) | **0.71 (0.58-0.86)** | 0.75 (0.53-1.08) | 0.042 |
| **Transport accidents** | | | | | | | | |
| Premature death | 458 | **1.93 (1.26-2.94)^†^** | 1.16 (0.83-1.64) | 0.96 (0.79-1.17) | 1.00 (0.82-1.22) | 0.90 (0.72-1.12) | 0.73 (0.47-1.13) | 0.047 |
| Death at or after the age of 70 | 147 | 0.59 (0.27-1.29) | 0.83 (0.48-1.45) | 0.69 (0.49-0.99) | 1.00 (0.71-1.41) | 0.73 (0.48-1.10) | 1.31 (0.66-2.62) | 0.347 |
| All death | 605 | 1.42 (0.97-2.06) | 1.09 (0.81-1.46) | 0.89 (0.75-1.06) | 1.00 (0.84-1.19) | 0.86 (0.71-1.04) | 0.85 (0.59-1.23) | 0.209 |
| **Falls** | | | | | | | | |
| Premature death | 109 | **4.88 (2.35-10.15)^†^** | 1.75 (0.88-3.49) | 1.26 (0.86-1.86) | 1.00 (0.63-1.59) | 1.00 (0.63-1.61) | 0.37 (0.13-1.03) | 0.005 |
| Death at or after the age of 70 | 353 | **2.06 (1.32-3.20)^†^** | **1.85 (1.30-2.62)** | 1.32 (1.07-1.63) | 1.00 (0.76-1.32) | 1.00 (0.77-1.31) | 0.96 (0.60-1.54) | 0.028 |
| All death | 462 | **2.51 (1.71-3.67)^†^** | **1.84 (1.35-2.51)** | 1.31 (1.09-1.58) | 1.00 (0.79-1.27) | 1.00 (0.80-1.26) | 0.79 (0.52-1.21) | 0.001 |
| **Intentional self-harm** | | | | | | | | |
| Premature death | 247 | **2.67 (1.49-4.77)^†^** | **2.04 (1.32-3.16)** | **1.60 (1.24-2.06)** | 1.00 (0.71-1.42) | 1.50 (1.11-2.01) | 1.13 (0.63-2.02) | 0.146 |
| Death at or after the age of 70 | 84 | 1.29 (0.58-2.90) | 0.68 (0.31-1.48) | 0.70 (0.44-1.11) | 1.00 (0.62-1.62) | 0.63 (0.35-1.13) | 0.95 (0.36-2.48) | 0.812 |
| All death | 331 | **2.27 (1.43-3.61)^†^** | 1.52 (1.04-2.23) | 1.29 (1.03-1.60) | 1.00 (0.75-1.33) | 1.20 (0.92-1.57) | 1.07 (0.65-1.77) | 0.167 |
| **Sequelae of other accidents** | | | | | | | | |
| Premature death | / | / | / | / | / | / | / | / |
| Death at or after the age of 70 | 83 | 1.45 (0.62-3.42) | 1.62 (0.87-3.01) | 1.00 (0.66-1.50) | 1.00 (0.57-1.75) | 1.02 (0.56-1.86) | 0.41 (0.11-1.58) | 0.209 |
| All death | 95 | 1.19 (0.53-2.69) | 1.39 (0.78-2.50) | 0.83 (0.56-1.24) | 1.00 (0.61-1.63) | 0.89 (0.51-1.56) | 0.34 (0.09-1.27) | 0.216 |
| **Less-common external causes of morbidity and mortality combined^*^** | | | | | | | | |
| Premature death | 239 | 1.16 (0.60-2.26) | 0.90 (0.54-1.49) | 0.82 (0.61-1.09) | 1.00 (0.77-1.30) | 0.94 (0.70-1.25) | 0.78 (0.44-1.39) | 0.798 |
| Death at or after the age of 70 | 176 | 0.78 (0.40-1.50) | 0.80 (0.47-1.35) | 0.77 (0.55-1.06) | 1.00 (0.72-1.40) | 0.92 (0.63-1.32) | 1.39 (0.74-2.64) | 0.261 |
| All death | 403 | 0.99 (0.61-1.59) | 0.86 (0.59-1.25) | 0.83 (0.66-1.03) | 1.00 (0.81-1.24) | 0.95 (0.76-1.20) | 1.03 (0.67-1.59) | 0.586 |

The models were adjusted for the same covariates as described in Figure 1. Linear trend tests were performed with the median value within each body mass index group. Bold indicates *P*<0.05.

^*^Included less-common ICD-10 codes within the corresponding ICD-10 chapter that were not individually investigated in the present study.

^†^FDR-adjusted *P*<0.05.

**Table S7. Summary of number of diseases associated with BMI by ICD-10 chapter after excluding those with relevant pre-existing diseases reported at baseline**

|  | **No. of diseases** | **<18.5 kg/m^2^** | | **18.5-19.9 kg/m^2^** | | **20.0-22.4 kg/m^2^** | | **24.0-27.9 kg/m^2^** | | **≥28.0 kg/m^2^** | |
| --- | --- | --- | --- | --- | --- | --- | --- | --- | --- | --- | --- |
|  |  | **Positive** | **Negative** | **Positive** | **Negative** | **Positive** | **Negative** | **Positive** | **Negative** | **Positive** | **Negative** |
| **Premature death** | | | | | | | | | | | |
| I Infectious and parasitic | 1 | 0 | 0 | 0 | 0 | 0 | 0 | 0 | 0 | 0 | 0 |
| II Neoplasms | 15 | 2 (0) | 0 | 0 | 1 (0) | 3 (0) | 0 | 0 | 0 | 0 | 0 |
| IV Endocrine, nutritional and metabolic | 1 | 1 (1) | 0 | 0 | 0 | 0 | 0 | 1 (0) | 0 | 1 (0) | 0 |
| IX Circulatory | 7 | 3 (3) | 0 | 1 (1) | 0 | 1 (1) | 0 | 0 | 0 | 1 (0) | 0 |
| X Respiratory | 2 | 1 (1) | 0 | 0 | 0 | 1 (0) | 0 | 0 | 0 | 0 | 0 |
| XI Digestive | 1 | 0 | 0 | 0 | 0 | 0 | 0 | 0 | 0 | 0 | 0 |
| XVIII Other symptoms, signs and abnormal findings | 1 | 0 | 0 | 0 | 0 | 0 | 0 | 0 | 0 | 0 | 0 |
| XX External causes | 4 | 3 (3) | 0 | 1 (0) | 0 | 1 (0) | 0 | 0 | 0 | 0 | 0 |
| Total | 32 | 10 (8) | 0 | 2 (1) | 1 (0) | 6 (1) | 0 | 1 (0) | 0 | 2 (0) | 0 |
| **Death at or after the age of 70** | | | | | | | | | | | |
| II Neoplasms | 13 | 3 (2) | 1 (0) | 1 (0) | 1 (0) | 0 | 0 | 0 | 0 | 0 | 0 |
| IV Endocrine, nutritional and metabolic | 2 | 0 | 0 | 0 | 0 | 0 | 0 | 0 | 0 | 0 | 0 |
| VI Nerve-related | 2 | 0 | 0 | 0 | 0 | 0 | 0 | 0 | 0 | 0 | 0 |
| IX Circulatory | 8 | 3 (2) | 0 | 1 (0) | 0 | 0 | 0 | 1 (0) | 1 (0) | 1 (0) | 0 |
| X Respiratory | 4 | 2 (2) | 0 | 0 | 0 | 0 | 0 | 0 | 1 (0) | 0 | 0 |
| XI Digestive | 2 | 0 | 0 | 1 (0) | 0 | 0 | 0 | 0 | 0 | 0 | 0 |
| XIV Genitourinary | 1 | 0 | 0 | 0 | 0 | 0 | 0 | 0 | 0 | 0 | 0 |
| XVIII Other symptoms, signs and abnormal findings | 2 | 0 | 0 | 0 | 0 | 0 | 0 | 0 | 1 (0) | 0 | 0 |
| XX External causes | 5 | 1 (1) | 0 | 1 (0) | 0 | 0 | 0 | 0 | 0 | 0 | 0 |
| Total | 39 | 9 (7) | 1 (0) | 4 (0) | 1 (0) | 0 | 0 | 1 (0) | 3 (0) | 1 (0) | 0 |
| **All death** | | | | | | | | | | | |
| I Infectious and parasitic | 2 | 1 (0) | 0 | 1 | 0 | 0 | 0 | 0 | 0 | 0 | 0 |
| II Neoplasms | 16 | 4 (3) | 1 (1) | 2 (0) | 0 | 2 (0) | 0 | 0 | 0 | 1 (0) | 0 |
| IV Endocrine, nutritional and metabolic | 2 | 2 (1) | 0 | 1 (0) | 0 | 0 | 0 | 0 | 0 | 0 | 0 |
| VI Nerve-related | 2 | 0 | 0 | 0 | 0 | 0 | 0 | 0 | 0 | 0 | 0 |
| IX Circulatory | 12 | 4 (3) | 0 | 2 (1) | 0 | 2 (0) | 0 | 0 | 1 (0) | 1 (0) | 0 |
| X Respiratory | 4 | 3 (2) | 0 | 0 | 0 | 0 | 0 | 0 | 1 (0) | 0 | 0 |
| XI Digestive | 2 | 0 | 0 | 1 (1) | 0 | 0 | 0 | 0 | 0 | 0 | 0 |
| XIV Genitourinary | 2 | 0 | 0 | 0 | 0 | 0 | 0 | 0 | 0 | 0 | 0 |
| XVIII Other symptoms, signs and abnormal findings | 2 | 1 (0) | 0 | 0 | 0 | 0 | 0 | 0 | 1 (0) | 0 | 0 |
| XX External causes | 5 | 2 (2) | 0 | 1 (1) | 0 | 0 | 0 | 0 | 0 | 0 | 0 |
| Total | 49 | 17 (11) | 1 (1) | 8 (3) | 0 | 4 (0) | 0 | 0 | 3 (0) | 2 (0) | 0 |

ICD-10, International Classification of Diseases, 10th Revision.

The group with a BMI of 22.5-23.9 kg/m^2^ was the reference group. The numbers in brackets are the results after adjusting for false discovery rate (FDR).

**Table S8. Summary of number of diseases associated with BMI by ICD-10 chapter after excluding those who died within the first five years of follow-up**

|  | **No. of diseases** | **<18.5 kg/m^2^** | | **18.5-19.9 kg/m^2^** | | **20.0-22.4 kg/m^2^** | | **24.0-27.9 kg/m^2^** | | **≥28.0 kg/m^2^** | |
| --- | --- | --- | --- | --- | --- | --- | --- | --- | --- | --- | --- |
|  |  | **Positive** | **Negative** | **Positive** | **Negative** | **Positive** | **Negative** | **Positive** | **Negative** | **Positive** | **Negative** |
| **Premature death** | | | | | | | | | | | |
| II Neoplasms | 13 | 0 | 0 | 0 | 0 | 2 (0) | 1 (0) | 0 | 0 | 2 (0) | 0 |
| IV Endocrine, nutritional and metabolic | 1 | 1 (1) | 0 | 1 (1) | 0 | 0 | 0 | 0 | 1 (0) | 0 | 1 (0) |
| IX Circulatory | 7 | 3 (2) | 0 | 1 (1) | 1 (0) | 2 (0) | 0 | 0 | 0 | 1 (0) | 0 |
| X Respiratory | 1 | 1 (1) | 0 | 0 | 0 | 1 (0) | 0 | 0 | 0 | 0 | 0 |
| XI Digestive | 1 | 0 | 0 | 0 | 0 | 0 | 0 | 0 | 0 | 0 | 0 |
| XVIII Other symptoms, signs and abnormal findings | 1 | 0 | 0 | 0 | 0 | 0 | 0 | 0 | 0 | 0 | 0 |
| XX External causes | 4 | 1 (0) | 0 | 0 | 0 | 0 | 0 | 0 | 0 | 0 | 0 |
| Total | 28 | 6 (4) | 0 | 2 (2) | 1 (0) | 5 (0) | 1 (0) | 0 | 1 (0) | 3 (0) | 1 (0) |
| **Death at or after the age of 70** | | | | | | | | | | | |
| II Neoplasms | 11 | 2 (2) | 1 (0) | 1 (0) | 1 (0) | 0 | 0 | 0 | 0 | 0 | 0 |
| IV Endocrine, nutritional and metabolic | 2 | 0 | 0 | 0 | 0 | 0 | 0 | 0 | 0 | 0 | 1 (0) |
| VI Nerve-related | 2 | 0 | 0 | 0 | 0 | 0 | 0 | 0 | 0 | 1 (0) | 0 |
| IX Circulatory | 8 | 3 (3) | 0 | 1 (0) | 0 | 0 | 0 | 0 | 1 (0) | 1 (0) | 0 |
| X Respiratory | 4 | 2 (2) | 0 | 0 | 0 | 0 | 0 | 0 | 0 | 0 | 0 |
| XI Digestive | 2 | 1 (0) | 0 | 1 (0) | 0 | 0 | 0 | 0 | 0 | 0 | 0 |
| XVIII Other symptoms, signs and abnormal findings | 2 | 0 | 0 | 0 | 0 | 0 | 0 | 0 | 1 (0) | 0 | 0 |
| XX External causes | 4 | 1 (1) | 0 | 1 (0) | 0 | 0 | 0 | 0 | 0 | 0 | 0 |
| Total | 35 | 9 (8) | 1 (0) | 4 (0) | 1 (0) | 0 | 0 | 0 | 2 (0) | 2 (0) | 1 (0) |
| **All death** | | | | | | | | | | | |
| I Infectious and parasitic | 2 | 0 | 0 | 0 | 0 | 0 | 0 | 0 | 0 | 0 | 0 |
| II Neoplasms | 15 | 3 (2) | 1 (0) | 0 | 0 | 2 (0) | 1 (0) | 1 (0) | 0 | 2 (0) | 0 |
| IV Endocrine, nutritional and metabolic | 2 | 2 (1) | 0 | 1 (0) | 0 | 0 | 0 | 0 | 1 (0) | 0 | 1 (1) |
| VI Nerve-related | 2 | 0 | 0 | 0 | 0 | 0 | 0 | 0 | 0 | 0 | 0 |
| IX Circulatory | 12 | 3 (3) | 0 | 1 (0) | 0 | 1 (0) | 0 | 0 | 0 | 1 (0) | 0 |
| X Respiratory | 4 | 3 (2) | 0 | 0 | 0 | 0 | 0 | 0 | 0 | 0 | 0 |
| XI Digestive | 2 | 1 (1) | 0 | 1 (0) | 0 | 1 (0) | 0 | 0 | 0 | 0 | 0 |
| XIV Genitourinary | 2 | 0 | 0 | 0 | 0 | 0 | 0 | 0 | 0 | 0 | 0 |
| XVIII Other symptoms, signs and abnormal findings | 2 | 0 | 0 | 0 | 0 | 0 | 0 | 0 | 1 (0) | 0 | 0 |
| XX External causes | 5 | 1 (1) | 0 | 1 (0) | 0 | 0 | 0 | 0 | 1 (0) | 0 | 0 |
| Total | 48 | 13 (10) | 1 (0) | 4 (0) | 0 | 4 (0) | 1 (0) | 1 (0) | 3 (0) | 3 (0) | 1 (1) |

ICD-10, International Classification of Diseases, 10th Revision.

The group with a BMI of 22.5-23.9 kg/m^2^ was the reference group. The numbers in brackets are the results after adjusting for false discovery rate (FDR).

**Table S9. Associations between BMI and cause-specific mortality** **after excluding those with relevant pre-existing diseases reported at baseline**

| **Disease** | **No. of deaths** | **<18.5 kg/m^2^** | **18.5-19.9 kg/m^2^** | **20.0-22.4 kg/m^2^** | **22.5-23.9 kg/m^2^** | **24.0-27.9 kg/m^2^** | **≥28.0 kg/m^2^** | ***P*_trend_** |
| --- | --- | --- | --- | --- | --- | --- | --- | --- |
| **Viral hepatitis (excluding 2,134 participants with hepatitis/cirrhosis at baseline)** | | | | | | | | |
| Premature death | 85 | 0.28 (0.04-2.16) | 0.41 (0.12-1.40) | 1.11 (0.67-1.83) | 1.00 (0.63-1.60) | 0.98 (0.64-1.51) | 1.09 (0.47-2.52) | 0.472 |
| Death at or after the age of 70 | / | / | / | / | / | / | / | / |
| All death | 131 | 0.69 (0.25-1.91) | 0.51 (0.21-1.22) | 0.92 (0.61-1.40) | 1.00 (0.68-1.48) | 1.16 (0.82-1.64) | 1.33 (0.68-2.57) | 0.186 |
| **Less-common certain infectious and parasitic diseases combined^*^ (excluding 2,487 participants with tuberculosis at baseline)** | | | | | | | | |
| Premature death | / | / | / | / | / | / | / | / |
| Death at or after the age of 70 | / | / | / | / | / | / | / | / |
| All death | 122 | **2.89 (1.37-6.07)** | **2.19 (1.23-3.87)** | 1.29 (0.91-1.84) | 1.00 (0.65-1.54) | 0.69 (0.43-1.10) | 0.66 (0.29-1.51) | 0.010 |
| **Type 2 diabetes mellitus (excluding 14,281 participants with diabetes at baseline)** | | | | | | | | |
| Premature death | 102 | **7.65 (2.66-22.04)^†^** | 2.44 (0.83-7.19) | 2.52 (1.41-4.48) | 1.00 (0.44-2.25) | **2.50 (1.87-3.33)** | **3.92 (2.20-6.98)** | 0.420 |
| Death at or after the age of 70 | 276 | 1.73 (0.89-3.36) | 0.52 (0.24-1.13) | 1.10 (0.79-1.53) | 1.00 (0.73-1.37) | 1.05 (0.86-1.29) | 1.19 (0.81-1.74) | 0.654 |
| All death | 378 | **2.37 (1.35-4.16)^†^** | 0.75 (0.40-1.41) | 1.30 (0.97-1.72) | 1.00 (0.75-1.34) | 1.25 (1.05-1.48) | 1.55 (1.13-2.13) | 0.407 |
| **Less-common diseases of the respiratory system combined^*^ (excluding 2,487 participants with tuberculosis at baseline)** | | | | | | | | |
| Premature death | / | / | / | / | / | / | / | / |
| Death at or after the age of 70 | 102 | 1.75 (0.72-4.27) | 0.82 (0.34-1.99) | 1.04 (0.65-1.67) | 1.00 (0.62-1.62) | 1.10 (0.72-1.67) | 1.05 (0.49-2.25) | 0.924 |
| All death | 140 | 2.59 (1.23-5.44) | 1.46 (0.75-2.83) | 1.43 (0.99-2.06) | 1.00 (0.65-1.54) | 1.05 (0.72-1.54) | 0.86 (0.43-1.72) | 0.135 |
| **Diseases of liver (excluding 2,134 participants with hepatitis/cirrhosis at baseline)** | | | | | | | | |
| Premature death | 100 | 1.57 (0.45-5.45) | 1.96 (0.93-4.17) | 1.40 (0.90-2.15) | 1.00 (0.64-1.57) | 0.72 (0.46-1.13) | 0.67 (0.30-1.50) | 0.057 |
| Death at or after the age of 70 | 112 | 2.75 (1.06-7.14) | **3.09 (1.62-5.89)** | 1.31 (0.84-2.06) | 1.00 (0.61-1.63) | 0.88 (0.58-1.34) | 0.86 (0.42-1.76) | 0.142 |
| All death | 212 | 2.14 (1.01-4.54) | **2.51 (1.54-4.09)^†^** | 1.36 (1.00-1.86) | 1.00 (0.72-1.39) | 0.80 (0.59-1.09) | 0.77 (0.45-1.31) | 0.008 |
| **Less-common diseases of the digestive system combined^*^ (excluding 24,253 participants with peptic ulcer or gallstones/gallbladder disease at baseline)** | | | | | | | | |
| Premature death | / | / | / | / | / | / | / | / |
| Death at or after the age of 70 | 202 | 0.74 (0.33-1.66) | 0.94 (0.53-1.65) | 1.02 (0.74-1.41) | 1.00 (0.73-1.36) | 0.89 (0.65-1.20) | 0.72 (0.40-1.27) | 0.539 |
| All death | 266 | 1.74 (0.97-3.15) | 1.19 (0.73-1.93) | 1.09 (0.83-1.44) | 1.00 (0.76-1.32) | 0.83 (0.63-1.09) | 0.60 (0.36-1.00) | 0.028 |
| **Chronic nephritic syndrome (excluding 3,741 participants with chronic kidney disease at baseline)** | | | | | | | | |
| Premature death | / | / | / | / | / | / | / | / |
| Death at or after the age of 70 | / | / | / | / | / | / | / | / |
| All death | 144 | 1.50 (0.70-3.21) | 1.69 (0.99-2.90) | 1.00 (0.70-1.43) | 1.00 (0.69-1.45) | 0.68 (0.45-1.04) | 0.78 (0.38-1.59) | 0.118 |
| **Less-common diseases of the genitourinary system combined^*^ (excluding 3,741 participants with chronic kidney disease at baseline)** | | | | | | | | |
| Premature death | / | / | / | / | / | / | / | / |
| Death at or after the age of 70 | / | / | / | / | / | / | / | / |
| All death | 153 | 2.26 (0.97-5.25) | 1.29 (0.62-2.68) | 1.10 (0.73-1.66) | 1.00 (0.67-1.48) | 1.03 (0.75-1.40) | 0.88 (0.49-1.60) | 0.345 |

The models were adjusted for the same covariates as described in Figure 1. Linear trend tests were performed with the median value within each body mass index group. Bold indicates *P*<0.05.

^*^Included less-common ICD-10 codes within the corresponding ICD-10 chapter that were not individually investigated in the present study.

^†^FDR-adjusted *P*<0.05.

**Table S10. Associations between BMI and cause-specific mortality** **after excluding those who died within the first five years of follow-up**

| **Disease** | **No. of deaths** | **<18.5 kg/m^2^** | **18.5-19.9 kg/m^2^** | **20.0-22.4 kg/m^2^** | **22.5-23.9 kg/m^2^** | **24.0-27.9 kg/m^2^** | **≥28.0 kg/m^2^** | ***P*_trend_** |
| --- | --- | --- | --- | --- | --- | --- | --- | --- |
| **Viral hepatitis** | | | | | | | | |
| Premature death | / | / | / | / | / | / | / | / |
| Death at or after the age of 70 | / | / | / | / | / | / | / | / |
| All death | 102 | 0.56 (0.18-1.76) | 0.46 (0.17-1.20) | 0.73 (0.44-1.19) | 1.00 (0.64-1.55) | 1.42 (0.97-2.08) | 1.44 (0.67-3.12) | 0.059 |
| **Less-common certain infectious and parasitic diseases combined^*^** | | | | | | | | |
| Premature death | / | / | / | / | / | / | / | / |
| Death at or after the age of 70 | / | / | / | / | / | / | / | / |
| All death | 102 | 2.32 (0.98-5.49) | 1.41 (0.69-2.87) | 0.98 (0.64-1.49) | 1.00 (0.66-1.52) | 0.64 (0.40-1.04) | 0.62 (0.26-1.48) | 0.081 |
| **Malignant neoplasm of oesophagus** | | | | | | | | |
| Premature death | 153 | 1.29 (0.44-3.77) | 1.26 (0.63-2.52) | 1.18 (0.80-1.74) | 1.00 (0.68-1.46) | 0.85 (0.62-1.17) | 0.95 (0.54-1.67) | 0.465 |
| Death at or after the age of 70 | 218 | 1.71 (0.99-2.95) | 1.01 (0.62-1.63) | 0.83 (0.62-1.12) | 1.00 (0.74-1.35) | 0.92 (0.67-1.26) | 0.74 (0.41-1.34) | 0.280 |
| All death | 371 | **1.74 (1.08-2.80)** | 1.13 (0.76-1.67) | 0.96 (0.76-1.22) | 1.00 (0.79-1.26) | 0.89 (0.71-1.12) | 0.88 (0.58-1.32) | 0.206 |
| **Malignant neoplasm of stomach** | | | | | | | | |
| Premature death | 306 | 0.83 (0.40-1.71) | 1.02 (0.64-1.63) | 0.95 (0.72-1.23) | 1.00 (0.78-1.28) | 0.99 (0.78-1.26) | 0.95 (0.60-1.51) | 0.945 |
| Death at or after the age of 70 | 345 | **2.94 (1.86-4.67)^†^** | **1.77 (1.18-2.66)** | 1.31 (1.01-1.68) | 1.00 (0.74-1.36) | 1.35 (1.06-1.70) | 1.48 (0.98-2.22) | 0.441 |
| All death | 651 | **1.79 (1.23-2.62)^†^** | 1.34 (0.98-1.82) | 1.10 (0.91-1.32) | 1.00 (0.82-1.21) | 1.14 (0.96-1.35) | 1.19 (0.88-1.61) | 0.600 |
| **Malignant neoplasm of colon and rectum** | | | | | | | | |
| Premature death | 311 | 1.40 (0.70-2.81) | 0.91 (0.52-1.58) | 1.38 (1.06-1.78) | 1.00 (0.77-1.30) | 1.04 (0.83-1.31) | 0.93 (0.59-1.46) | 0.359 |
| Death at or after the age of 70 | 384 | 1.21 (0.71-2.06) | 0.64 (0.38-1.08) | 0.96 (0.74-1.24) | 1.00 (0.78-1.28) | 1.10 (0.90-1.34) | 1.26 (0.88-1.81) | 0.234 |
| All death | 695 | 1.31 (0.86-1.99) | 0.74 (0.51-1.09) | 1.14 (0.95-1.37) | 1.00 (0.84-1.20) | 1.08 (0.93-1.25) | 1.13 (0.85-1.49) | 0.728 |
| **Malignant neoplasm of liver and intrahepatic bile ducts** | | | | | | | | |
| Premature death | 408 | 1.53 (0.90-2.57) | 1.15 (0.77-1.74) | 1.21 (0.97-1.52) | 1.00 (0.78-1.28) | 1.27 (1.03-1.56) | **1.62 (1.10-2.38)** | 0.396 |
| Death at or after the age of 70 | 384 | 1.18 (0.69-2.03) | 1.48 (1.01-2.19) | 0.97 (0.74-1.25) | 1.00 (0.77-1.30) | 1.29 (1.05-1.58) | 1.51 (1.04-2.19) | 0.305 |
| All death | 792 | 1.33 (0.91-1.94) | 1.33 (1.00-1.76) | 1.11 (0.93-1.31) | 1.00 (0.84-1.20) | **1.28 (1.10-1.48)** | **1.55 (1.19-2.03)** | 0.197 |
| **Malignant neoplasm of gallbladder and biliary tract** | | | | | | | | |
| Premature death | 95 | 0.96 (0.27-3.39) | 1.36 (0.63-2.93) | 1.05 (0.67-1.67) | 1.00 (0.64-1.57) | 0.90 (0.57-1.42) | 1.05 (0.45-2.44) | 0.769 |
| Death at or after the age of 70 | 124 | 1.06 (0.45-2.54) | 0.99 (0.49-1.98) | 0.76 (0.49-1.20) | 1.00 (0.66-1.52) | 0.97 (0.66-1.44) | 1.44 (0.74-2.80) | 0.388 |
| All death | 219 | 1.06 (0.52-2.14) | 1.15 (0.68-1.92) | 0.89 (0.65-1.23) | 1.00 (0.74-1.36) | 0.93 (0.69-1.25) | 1.24 (0.74-2.10) | 0.698 |
| **Malignant neoplasm of pancreas** | | | | | | | | |
| Premature death | 184 | 1.54 (0.70-3.38) | 0.51 (0.22-1.20) | 1.13 (0.79-1.61) | 1.00 (0.70-1.44) | 1.51 (1.14-2.01) | 1.68 (0.95-2.98) | 0.143 |
| Death at or after the age of 70 | 250 | 1.04 (0.49-2.22) | 1.07 (0.62-1.86) | 0.70 (0.49-0.99) | 1.00 (0.76-1.31) | 0.80 (0.63-1.02) | 0.68 (0.43-1.08) | 0.430 |
| All death | 434 | 1.23 (0.71-2.13) | 0.83 (0.53-1.32) | 0.87 (0.68-1.12) | 1.00 (0.80-1.24) | 1.03 (0.85-1.24) | 0.96 (0.67-1.38) | 0.815 |
| **Malignant neoplasm of trachea, bronchus and lung** | | | | | | | | |
| Premature death | 759 | 1.18 (0.76-1.83) | 1.18 (0.88-1.60) | **1.37 (1.17-1.60)** | 1.00 (0.84-1.18) | 1.02 (0.87-1.20) | 1.15 (0.85-1.55) | 0.391 |
| Death at or after the age of 70 | 768 | 1.08 (0.74-1.57) | 1.04 (0.77-1.40) | 1.15 (0.98-1.35) | 1.00 (0.85-1.18) | 0.86 (0.73-1.01) | 0.76 (0.57-1.02) | 0.049 |
| All death | 1,527 | 1.13 (0.85-1.50) | 1.12 (0.90-1.38) | **1.26 (1.13-1.41)** | 1.00 (0.89-1.13) | 0.94 (0.84-1.05) | 0.93 (0.75-1.14) | 0.041 |
| **Malignant neoplasm of breast** | | | | | | | | |
| Premature death | 325 | 1.27 (0.68-2.37) | 0.71 (0.42-1.22) | 0.93 (0.71-1.22) | 1.00 (0.78-1.29) | 1.06 (0.85-1.32) | **1.76 (1.18-2.62)** | 0.045 |
| Death at or after the age of 70 | 129 | 1.97 (0.73-5.33) | 0.79 (0.27-2.29) | 1.30 (0.79-2.15) | 1.00 (0.59-1.70) | 1.59 (1.21-2.09) | 1.96 (1.14-3.37) | 0.250 |
| All death | 454 | 1.42 (0.83-2.40) | 0.74 (0.46-1.19) | 1.01 (0.80-1.29) | 1.00 (0.80-1.26) | 1.16 (0.98-1.39) | **1.75 (1.27-2.42)** | 0.025 |
| **Malignant neoplasm of cervix uteri** | | | | | | | | |
| Premature death | 167 | 1.23 (0.47-3.21) | 1.62 (0.91-2.88) | **1.70 (1.25-2.30)** | 1.00 (0.69-1.46) | 0.99 (0.69-1.43) | 0.93 (0.47-1.84) | 0.144 |
| Death at or after the age of 70 | 90 | 1.14 (0.42-3.07) | 0.82 (0.34-1.98) | 1.49 (1.00-2.22) | 1.00 (0.59-1.70) | 1.07 (0.64-1.78) | 0.92 (0.35-2.40) | 0.725 |
| All death | 257 | 1.24 (0.63-2.44) | 1.30 (0.80-2.09) | **1.64 (1.29-2.09)** | 1.00 (0.74-1.36) | 1.02 (0.76-1.37) | 0.93 (0.53-1.62) | 0.162 |
| **Malignant neoplasm of corpus uteri** | | | | | | | | |
| Premature death | / | / | / | / | / | / | / | / |
| Death at or after the age of 70 | / | / | / | / | / | / | / | / |
| All death | 85 | 1.15 (0.36-3.69) | 0.68 (0.23-1.99) | 1.07 (0.63-1.81) | 1.00 (0.58-1.73) | 1.49 (0.98-2.24) | 2.21 (1.02-4.80) | 0.130 |
| **Malignant neoplasm of uterus, part unspecified** | | | | | | | | |
| Premature death | / | / | / | / | / | / | / | / |
| Death at or after the age of 70 | / | / | / | / | / | / | / | / |
| All death | 90 | 2.42 (0.91-6.45) | 2.17 (1.05-4.48) | 1.32 (0.84-2.09) | 1.00 (0.58-1.73) | 1.07 (0.66-1.74) | 1.25 (0.54-2.91) | 0.412 |
| **Malignant neoplasm of ovary** | | | | | | | | |
| Premature death | 135 | 0.78 (0.23-2.64) | 0.91 (0.42-1.97) | 1.29 (0.88-1.89) | 1.00 (0.67-1.49) | 1.19 (0.83-1.70) | 0.98 (0.47-2.02) | 0.989 |
| Death at or after the age of 70 | / | / | / | / | / | / | / | / |
| All death | 207 | 1.15 (0.49-2.65) | 0.94 (0.50-1.76) | 1.22 (0.89-1.67) | 1.00 (0.73-1.38) | 1.05 (0.79-1.41) | 0.96 (0.55-1.68) | 0.720 |
| **Malignant neoplasms of central nervous system** | | | | | | | | |
| Premature death | 96 | 0.17 (0.02-1.31) | 0.45 (0.17-1.20) | 0.95 (0.61-1.50) | 1.00 (0.65-1.53) | 0.89 (0.57-1.40) | 2.39 (1.12-5.09) | 0.030 |
| Death at or after the age of 70 | / | / | / | / | / | / | / | / |
| All death | 169 | 0.49 (0.20-1.22) | 0.66 (0.35-1.23) | 0.74 (0.52-1.06) | 1.00 (0.73-1.37) | 0.92 (0.66-1.28) | 1.44 (0.79-2.63) | 0.085 |
| **Malignant neoplasms, stated or presumed to be primary, of lymphoid, haematopoietic and related tissue** | | | | | | | | |
| Premature death | 220 | 0.60 (0.25-1.44) | 1.17 (0.72-1.89) | **0.65 (0.47-0.90)** | 1.00 (0.77-1.31) | 0.85 (0.63-1.13) | 1.11 (0.64-1.91) | 0.511 |
| Death at or after the age of 70 | 191 | **0.28 (0.10-0.82)** | **0.47 (0.23-0.94)** | 0.81 (0.57-1.15) | 1.00 (0.75-1.34) | 0.78 (0.58-1.05) | 1.19 (0.70-2.02) | 0.115 |
| All death | 411 | **0.42 (0.21-0.83)** | 0.83 (0.56-1.22) | **0.73 (0.57-0.92)** | 1.00 (0.82-1.22) | 0.81 (0.66-1.00) | 1.13 (0.77-1.65) | 0.133 |
| **Less-common neoplasms combined^*^** | | | | | | | | |
| Premature death | 388 | 1.14 (0.63-2.06) | 1.19 (0.79-1.79) | 0.90 (0.71-1.16) | 1.00 (0.80-1.26) | 1.02 (0.83-1.26) | 1.27 (0.86-1.87) | 0.546 |
| Death at or after the age of 70 | 475 | **2.37 (1.55-3.62)^†^** | 0.74 (0.46-1.20) | 1.21 (0.97-1.50) | 1.00 (0.80-1.24) | 0.94 (0.78-1.14) | 0.92 (0.65-1.30) | 0.129 |
| All death | 616 | **1.82 (1.22-2.71)^†^** | 0.86 (0.59-1.25) | 1.02 (0.84-1.24) | 1.00 (0.83-1.20) | 0.92 (0.78-1.09) | 0.95 (0.70-1.29) | 0.364 |
| **Type 2 diabetes mellitus** | | | | | | | | |
| Premature death | 339 | **3.80 (2.14-6.75)^†^** | **2.42 (1.56-3.75)^†^** | 1.40 (1.08-1.83) | 1.00 (0.77-1.30) | **0.68 (0.55-0.85)** | **0.51 (0.35-0.77)** | <0.001 |
| Death at or after the age of 70 | 690 | 1.45 (0.93-2.25) | 0.72 (0.47-1.09) | 1.13 (0.93-1.37) | 1.00 (0.84-1.19) | 0.80 (0.70-0.93) | **0.67 (0.51-0.87)** | 0.010 |
| All death | 1,029 | **1.99 (1.40-2.83)^†^** | 1.14 (0.84-1.55) | 1.22 (1.04-1.42) | 1.00 (0.86-1.16) | **0.76 (0.68-0.86)** | **0.61 (0.49-0.76)^†^** | <0.001 |
| **Less-common endocrine, nutritional and metabolic diseases combined^*^** | | | | | | | | |
| Premature death | / | / | / | / | / | / | / | / |
| Death at or after the age of 70 | 109 | 1.38 (0.51-3.79) | 1.43 (0.68-2.99) | 0.98 (0.61-1.58) | 1.00 (0.65-1.53) | 0.70 (0.46-1.06) | 0.79 (0.39-1.61) | 0.326 |
| All death | 149 | **2.42 (1.10-5.34)** | **2.21 (1.24-3.93)** | 0.94 (0.62-1.43) | 1.00 (0.68-1.47) | 0.69 (0.48-0.99) | 0.85 (0.47-1.57) | 0.100 |
| **Other degenerative diseases of nervous system, not elsewhere classified** | | | | | | | | |
| Premature death | / | / | / | / | / | / | / | / |
| Death at or after the age of 70 | 108 | 1.10 (0.54-2.26) | 1.07 (0.59-1.95) | 1.24 (0.89-1.73) | 1.00 (0.58-1.74) | 1.39 (0.82-2.38) | **2.80 (1.13-6.94)** | 0.232 |
| All death | 125 | 1.03 (0.51-2.06) | 1.00 (0.57-1.75) | 1.01 (0.72-1.40) | 1.00 (0.63-1.59) | 1.20 (0.74-1.93) | 2.08 (0.90-4.81) | 0.302 |
| **Less-common diseases of the nervous system combined^*^** | | | | | | | | |
| Premature death | / | / | / | / | / | / | / | / |
| Death at or after the age of 70 | 154 | 1.07 (0.53-2.14) | 1.27 (0.76-2.15) | 0.79 (0.56-1.12) | 1.00 (0.70-1.43) | 0.96 (0.65-1.42) | 0.75 (0.34-1.63) | 0.610 |
| All death | 235 | 1.19 (0.66-2.15) | 1.34 (0.87-2.06) | 0.99 (0.76-1.30) | 1.00 (0.73-1.36) | 1.20 (0.88-1.65) | 0.87 (0.46-1.66) | 0.725 |
| **Chronic rheumatic heart disease** | | | | | | | | |
| Premature death | / | / | / | / | / | / | / | / |
| Death at or after the age of 70 | / | / | / | / | / | / | / | / |
| All death | 82 | 2.18 (0.94-5.10) | 0.33 (0.10-1.07) | 0.78 (0.48-1.27) | 1.00 (0.63-1.59) | 0.79 (0.47-1.34) | 0.90 (0.34-2.36) | 0.750 |
| **Hypertensive diseases** | | | | | | | | |
| Premature death | 98 | 0.84 (0.24-2.96) | 0.84 (0.34-2.06) | 0.75 (0.45-1.25) | 1.00 (0.66-1.52) | 0.67 (0.44-1.02) | 1.37 (0.68-2.74) | 0.420 |
| Death at or after the age of 70 | 739 | 1.05 (0.74-1.48) | 0.88 (0.65-1.17) | 0.92 (0.78-1.09) | 1.00 (0.84-1.19) | 1.14 (0.97-1.34) | 1.25 (0.93-1.68) | 0.134 |
| All death | 837 | 1.03 (0.74-1.44) | 0.87 (0.66-1.15) | 0.89 (0.76-1.05) | 1.00 (0.85-1.17) | 1.07 (0.92-1.24) | 1.29 (0.98-1.70) | 0.085 |
| **Ischaemic heart diseases other than myocardial infarction** | | | | | | | | |
| Premature death | 263 | **2.88 (1.51-5.51)^†^** | 1.13 (0.61-2.11) | 1.02 (0.73-1.42) | 1.00 (0.74-1.35) | 0.77 (0.61-0.98) | 1.20 (0.80-1.79) | 0.711 |
| Death at or after the age of 70 | 1,562 | **1.63 (1.25-2.11)^†^** | 1.25 (1.00-1.55) | 1.19 (1.05-1.35) | 1.00 (0.88-1.14) | 1.09 (0.99-1.21) | 1.13 (0.95-1.35) | 0.366 |
| All death | 1,825 | **1.75 (1.37-2.23)^†^** | 1.24 (1.01-1.53) | 1.16 (1.03-1.31) | 1.00 (0.89-1.13) | 1.04 (0.95-1.14) | 1.15 (0.98-1.35) | 0.334 |
| **Myocardial infarction** | | | | | | | | |
| Premature death | 694 | **3.39 (2.32-4.95)^†^** | **1.78 (1.28-2.47)^†^** | **1.50 (1.25-1.81)** | 1.00 (0.81-1.23) | 1.29 (1.11-1.49) | 1.20 (0.91-1.58) | 0.024 |
| Death at or after the age of 70 | 1,869 | **1.57 (1.27-1.94)^†^** | 1.17 (0.98-1.40) | 1.08 (0.97-1.19) | 1.00 (0.90-1.12) | **0.84 (0.75-0.93)** | 0.97 (0.81-1.16) | 0.011 |
| All death | 2,563 | **1.89 (1.58-2.27)^†^** | **1.31 (1.12-1.53)** | **1.18 (1.07-1.29)** | 1.00 (0.91-1.10) | 0.94 (0.86-1.02) | 1.02 (0.87-1.18) | 0.001 |
| **Cardiac arrest** | | | | | | | | |
| Premature death | / | / | / | / | / | / | / | / |
| Death at or after the age of 70 | / | / | / | / | / | / | / | / |
| All death | 82 | 2.14 (0.77-5.94) | 1.10 (0.42-2.86) | 1.34 (0.84-2.12) | 1.00 (0.60-1.65) | 0.62 (0.36-1.04) | 1.29 (0.58-2.88) | 0.554 |
| **Subarachnoid haemorrhage** | | | | | | | | |
| Premature death | / | / | / | / | / | / | / | / |
| Death at or after the age of 70 | / | / | / | / | / | / | / | / |
| All death | 108 | 0.94 (0.26-3.38) | 0.82 (0.31-2.14) | 1.18 (0.74-1.89) | 1.00 (0.63-1.58) | 1.21 (0.85-1.74) | 0.93 (0.44-1.96) | 0.999 |
| **Intracerebral haemorrhage** | | | | | | | | |
| Premature death | 962 | **1.49 (1.07-2.07)** | **0.69 (0.50-0.95)** | 0.91 (0.78-1.07) | 1.00 (0.86-1.16) | 1.05 (0.92-1.19) | **1.37 (1.08-1.73)** | 0.058 |
| Death at or after the age of 70 | 1,567 | **1.45 (1.18-1.80)^†^** | **1.34 (1.13-1.58)** | 1.00 (0.90-1.11) | 1.00 (0.88-1.13) | 1.02 (0.90-1.16) | 0.88 (0.70-1.11) | 0.017 |
| All death | 2,529 | **1.44 (1.20-1.72)^†^** | 1.12 (0.97-1.30) | 0.96 (0.88-1.05) | 1.00 (0.91-1.10) | 1.03 (0.94-1.13) | 1.09 (0.92-1.28) | 0.528 |
| **Cerebral infarction** | | | | | | | | |
| Premature death | 269 | 1.30 (0.58-2.93) | 1.31 (0.74-2.29) | **1.52 (1.14-2.03)** | 1.00 (0.73-1.36) | 0.97 (0.76-1.23) | 1.26 (0.82-1.92) | 0.588 |
| Death at or after the age of 70 | 1,082 | 1.08 (0.80-1.45) | 1.09 (0.87-1.38) | 0.89 (0.77-1.03) | 1.00 (0.87-1.15) | 0.97 (0.85-1.11) | 1.17 (0.93-1.47) | 0.472 |
| All death | 1,351 | 1.15 (0.87-1.51) | 1.15 (0.93-1.42) | 0.99 (0.87-1.13) | 1.00 (0.88-1.14) | 0.97 (0.86-1.09) | 1.20 (0.98-1.47) | 0.650 |
| **Stroke, not specified as haemorrhage or infarction** | | | | | | | | |
| Premature death | / | / | / | / | / | / | / | / |
| Death at or after the age of 70 | 99 | 0.47 (0.15-1.45) | 0.94 (0.45-1.93) | 1.09 (0.72-1.66) | 1.00 (0.63-1.58) | 1.03 (0.65-1.64) | 1.35 (0.59-3.10) | 0.462 |
| All death | 126 | 0.55 (0.20-1.52) | 0.74 (0.36-1.50) | 0.87 (0.59-1.28) | 1.00 (0.71-1.42) | 0.66 (0.44-0.99) | 0.81 (0.39-1.66) | 0.914 |
| **Other cerebrovascular diseases** | | | | | | | | |
| Premature death | / | / | / | / | / | / | / | / |
| Death at or after the age of 70 | / | / | / | / | / | / | / | / |
| All death | 89 | 1.52 (0.47-4.89) | 1.55 (0.65-3.67) | 1.06 (0.61-1.83) | 1.00 (0.59-1.69) | 1.26 (0.83-1.91) | 0.79 (0.34-1.81) | 0.537 |
| **Sequelae of cerebrovascular disease** | | | | | | | | |
| Premature death | 312 | 0.70 (0.27-1.78) | 0.55 (0.27-1.10) | 0.98 (0.72-1.33) | 1.00 (0.76-1.31) | 0.91 (0.75-1.10) | 1.01 (0.70-1.46) | 0.542 |
| Death at or after the age of 70 | 1,303 | 0.77 (0.55-1.08) | 1.17 (0.93-1.47) | 1.00 (0.87-1.15) | 1.00 (0.87-1.15) | 1.19 (1.07-1.33) | **1.41 (1.16-1.71)** | 0.012 |
| All death | 1,615 | 0.77 (0.56-1.05) | 1.07 (0.86-1.33) | 1.00 (0.88-1.13) | 1.00 (0.89-1.13) | 1.14 (1.03-1.25) | **1.34 (1.13-1.59)** | 0.010 |
| **Less-common diseases of the circulatory system combined^*^** | | | | | | | | |
| Premature death | 217 | 1.04 (0.43-2.51) | 0.82 (0.43-1.57) | 0.93 (0.68-1.29) | 1.00 (0.76-1.32) | 0.72 (0.54-0.95) | 0.68 (0.40-1.15) | 0.299 |
| Death at or after the age of 70 | 428 | 1.51 (0.97-2.35) | 0.92 (0.62-1.39) | 0.92 (0.73-1.17) | 1.00 (0.80-1.25) | 0.95 (0.77-1.17) | 1.21 (0.85-1.74) | 0.734 |
| All death | 339 | 1.42 (0.86-2.34) | 0.86 (0.54-1.34) | 0.82 (0.63-1.06) | 1.00 (0.79-1.27) | 0.86 (0.68-1.09) | 1.21 (0.80-1.82) | 0.631 |
| **Pneumonia** | | | | | | | | |
| Premature death | / | / | / | / | / | / | / | / |
| Death at or after the age of 70 | 379 | **2.64 (1.62-4.31)^†^** | 1.52 (0.99-2.32) | 1.21 (0.95-1.55) | 1.00 (0.78-1.28) | 0.73 (0.58-0.91) | 0.76 (0.52-1.11) | 0.003 |
| All death | 454 | **2.63 (1.66-4.16)^†^** | 1.50 (1.01-2.23) | 1.14 (0.90-1.44) | 1.00 (0.80-1.25) | 0.77 (0.64-0.94) | 0.77 (0.55-1.08) | 0.003 |
| **Chronic obstructive pulmonary disease** | | | | | | | | |
| Premature death | 134 | **7.46 (3.92-14.19)^†^** | 1.93 (0.96-3.90) | **2.25 (1.63-3.11)** | 1.00 (0.61-1.64) | 1.13 (0.73-1.74) | 0.92 (0.42-2.02) | 0.003 |
| Death at or after the age of 70 | 780 | **2.20 (1.71-2.83)^†^** | 1.17 (0.93-1.47) | 0.99 (0.87-1.14) | 1.00 (0.83-1.20) | 0.84 (0.69-1.03) | 1.09 (0.77-1.53) | 0.009 |
| All death | 914 | **2.61 (2.07-3.30)^†^** | 1.28 (1.03-1.59) | 1.12 (0.99-1.27) | 1.00 (0.84-1.19) | 0.88 (0.73-1.06) | 1.06 (0.77-1.45) | <0.001 |
| **Other respiratory disorders** | | | | | | | | |
| Premature death | / | / | / | / | / | / | / | / |
| Death at or after the age of 70 | 87 | 2.25 (0.74-6.80) | 1.83 (0.76-4.39) | 1.21 (0.69-2.11) | 1.00 (0.54-1.84) | 1.31 (0.87-1.97) | 1.60 (0.78-3.28) | 0.921 |
| All death | 106 | **3.19 (1.28-7.92)** | 1.54 (0.66-3.56) | 1.35 (0.83-2.19) | 1.00 (0.58-1.74) | 1.34 (0.92-1.97) | 1.86 (0.96-3.60) | 0.862 |
| **Less-common diseases of the respiratory system combined^*^** | | | | | | | | |
| Premature death | / | / | / | / | / | / | / | / |
| Death at or after the age of 70 | 99 | 1.51 (0.60-3.82) | 0.81 (0.33-1.97) | 0.98 (0.61-1.58) | 1.00 (0.63-1.60) | 0.92 (0.60-1.42) | 0.94 (0.43-2.03) | 0.802 |
| All death | 127 | 2.26 (1.03-4.93) | 1.32 (0.66-2.66) | 1.25 (0.85-1.84) | 1.00 (0.65-1.54) | 0.94 (0.63-1.41) | 0.78 (0.37-1.63) | 0.166 |
| **Diseases of liver** | | | | | | | | |
| Premature death | 83 | 2.89 (0.81-10.27) | 1.83 (0.70-4.81) | 1.99 (1.24-3.22) | 1.00 (0.57-1.77) | 0.98 (0.62-1.56) | 0.90 (0.38-2.10) | 0.115 |
| Death at or after the age of 70 | 104 | **3.67 (1.40-9.63)** | **3.75 (1.92-7.31)** | 1.60 (1.01-2.52) | 1.00 (0.58-1.73) | 1.02 (0.66-1.57) | 0.97 (0.46-2.04) | 0.047 |
| All death | 187 | **3.25 (1.51-6.96)^†^** | **2.86 (1.67-4.91)** | **1.78 (1.28-2.46)** | 1.00 (0.67-1.48) | 1.00 (0.73-1.38) | 0.96 (0.55-1.67) | 0.013 |
| **Less-common diseases of the digestive system combined^*^** | | | | | | | | |
| Premature death | / | / | / | / | / | / | / | / |
| Death at or after the age of 70 | 241 | 1.09 (0.56-2.10) | 0.97 (0.58-1.64) | 1.22 (0.93-1.61) | 1.00 (0.74-1.34) | 0.88 (0.66-1.18) | 0.82 (0.48-1.39) | 0.343 |
| All death | 302 | 1.78 (1.03-3.08) | 1.09 (0.68-1.76) | 1.30 (1.02-1.66) | 1.00 (0.76-1.31) | 0.87 (0.67-1.13) | 0.71 (0.44-1.14) | 0.036 |
| **Chronic nephritic syndrome** | | | | | | | | |
| Premature death | / | / | / | / | / | / | / | / |
| Death at or after the age of 70 | / | / | / | / | / | / | / | / |
| All death | 129 | 1.63 (0.76-3.48) | 1.25 (0.68-2.30) | 0.86 (0.58-1.26) | 1.00 (0.69-1.44) | 0.59 (0.38-0.92) | 0.76 (0.36-1.59) | 0.195 |
| **Less-common diseases of the genitourinary system combined^*^** | | | | | | | | |
| Premature death | / | / | / | / | / | / | / | / |
| Death at or after the age of 70 | / | / | / | / | / | / | / | / |
| All death | 136 | 2.44 (0.94-6.34) | 1.42 (0.62-3.24) | 1.40 (0.90-2.17) | 1.00 (0.63-1.59) | 1.31 (0.96-1.79) | 1.09 (0.59-2.00) | 0.502 |
| **Senility** | | | | | | | | |
| Premature death | / | / | / | / | / | / | / | / |
| Death at or after the age of 70 | 108 | 1.20 (0.51-2.83) | 1.98 (1.11-3.52) | 1.16 (0.79-1.69) | 1.00 (0.63-1.59) | 0.81 (0.49-1.33) | 0.51 (0.20-1.32) | 0.071 |
| All death | 109 | 1.13 (0.48-2.66) | 1.91 (1.07-3.40) | 1.13 (0.77-1.66) | 1.00 (0.63-1.58) | 0.83 (0.51-1.36) | 0.62 (0.25-1.52) | 0.132 |
| **Ill-defined and unknown causes of mortality** | | | | | | | | |
| Premature death | 156 | 1.13 (0.39-3.31) | 1.19 (0.59-2.39) | 1.12 (0.77-1.62) | 1.00 (0.71-1.40) | 0.76 (0.55-1.06) | 0.62 (0.33-1.18) | 0.130 |
| Death at or after the age of 70 | 259 | 1.23 (0.65-2.32) | 0.95 (0.57-1.58) | 0.82 (0.61-1.10) | 1.00 (0.78-1.28) | **0.61 (0.46-0.80)** | 0.66 (0.41-1.06) | 0.145 |
| All death | 415 | 1.26 (0.74-2.16) | 1.02 (0.68-1.55) | 0.92 (0.73-1.16) | 1.00 (0.82-1.22) | **0.67 (0.54-0.82)** | 0.66 (0.45-0.96) | 0.041 |
| **Transport accidents** | | | | | | | | |
| Premature death | 305 | 1.28 (0.71-2.29) | 1.23 (0.82-1.85) | 0.95 (0.75-1.21) | 1.00 (0.79-1.27) | 0.74 (0.56-0.97) | 0.85 (0.51-1.43) | 0.188 |
| Death at or after the age of 70 | 125 | 0.86 (0.35-2.08) | 1.45 (0.82-2.55) | 0.91 (0.63-1.32) | 1.00 (0.67-1.48) | 0.69 (0.44-1.10) | 0.98 (0.46-2.12) | 0.577 |
| All death | 430 | 1.10 (0.67-1.80) | 1.31 (0.94-1.82) | 0.94 (0.77-1.15) | 1.00 (0.82-1.23) | **0.72 (0.57-0.92)** | 0.89 (0.58-1.36) | 0.157 |
| **Falls** | | | | | | | | |
| Premature death | 83 | **3.11 (1.25-7.74)** | 1.54 (0.71-3.36) | 1.13 (0.72-1.77) | 1.00 (0.60-1.66) | 1.04 (0.61-1.77) | 0.36 (0.11-1.24) | 0.066 |
| Death at or after the age of 70 | 342 | **2.18 (1.39-3.41)^†^** | **1.75 (1.21-2.51)** | 1.33 (1.07-1.65) | 1.00 (0.75-1.32) | 1.02 (0.78-1.34) | 1.00 (0.63-1.61) | 0.043 |
| All death | 425 | **2.30 (1.54-3.44)^†^** | **1.71 (1.23-2.37)** | 1.28 (1.06-1.56) | 1.00 (0.78-1.28) | 1.02 (0.81-1.30) | 0.86 (0.55-1.33) | 0.008 |
| **Intentional self-harm** | | | | | | | | |
| Premature death | 167 | 2.19 (1.03-4.69) | 1.50 (0.83-2.70) | 1.39 (1.01-1.93) | 1.00 (0.68-1.47) | 1.19 (0.84-1.68) | 1.04 (0.54-2.03) | 0.317 |
| Death at or after the age of 70 | / | / | / | / | / | / | / | / |
| All death | 235 | 1.88 (1.06-3.33) | 1.04 (0.63-1.73) | 1.04 (0.78-1.37) | 1.00 (0.74-1.35) | 0.97 (0.72-1.32) | 1.01 (0.58-1.77) | 0.482 |
| **Sequelae of other accidents** | | | | | | | | |
| Premature death | / | / | / | / | / | / | / | / |
| Death at or after the age of 70 | 82 | 1.53 (0.65-3.60) | 1.55 (0.82-2.94) | 1.01 (0.67-1.52) | 1.00 (0.57-1.75) | 1.00 (0.55-1.83) | 0.39 (0.10-1.53) | 0.194 |
| All death | 93 | 1.14 (0.49-2.64) | 1.36 (0.74-2.48) | 0.84 (0.57-1.26) | 1.00 (0.61-1.63) | 0.87 (0.49-1.52) | 0.32 (0.08-1.21) | 0.210 |
| **Less-common external causes of morbidity and mortality combined^*^** | | | | | | | | |
| Premature death | 158 | 0.83 (0.33-2.06) | 0.76 (0.39-1.48) | 0.79 (0.55-1.14) | 1.00 (0.72-1.38) | 0.99 (0.70-1.39) | 0.80 (0.40-1.61) | 0.770 |
| Death at or after the age of 70 | 167 | 0.76 (0.38-1.52) | 0.88 (0.52-1.49) | 0.78 (0.56-1.08) | 1.00 (0.71-1.41) | 0.84 (0.57-1.23) | 1.34 (0.70-2.57) | 0.397 |
| All death | 314 | 0.85 (0.49-1.48) | 0.83 (0.54-1.27) | 0.81 (0.63-1.05) | 1.00 (0.78-1.27) | 0.95 (0.74-1.23) | 1.11 (0.69-1.78) | 0.402 |

The models were adjusted for the same covariates as described in Figure 1. Linear trend tests were performed with the median value within each body mass index group. Bold indicates *P*<0.05.

^*^Included less-common ICD-10 codes within the corresponding ICD-10 chapter that were not individually investigated in the present study.

^†^FDR-adjusted *P*<0.05.


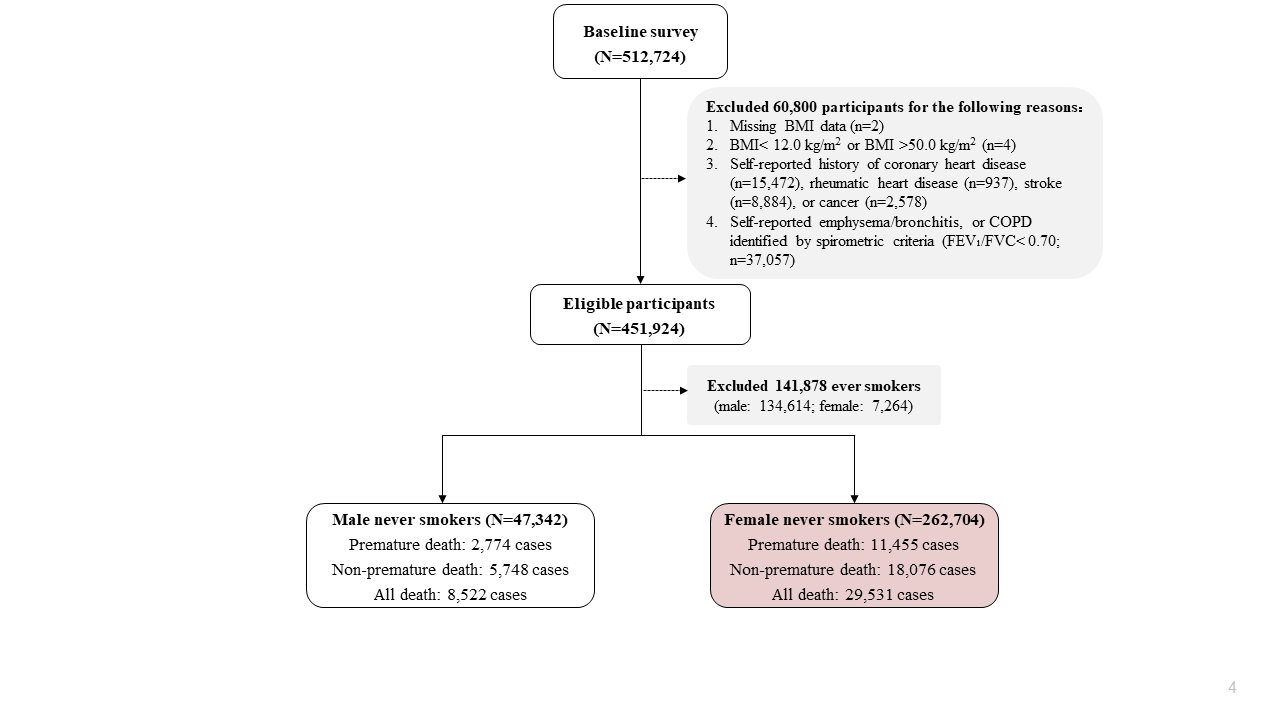


**Figure S1. The flowchart of participants**


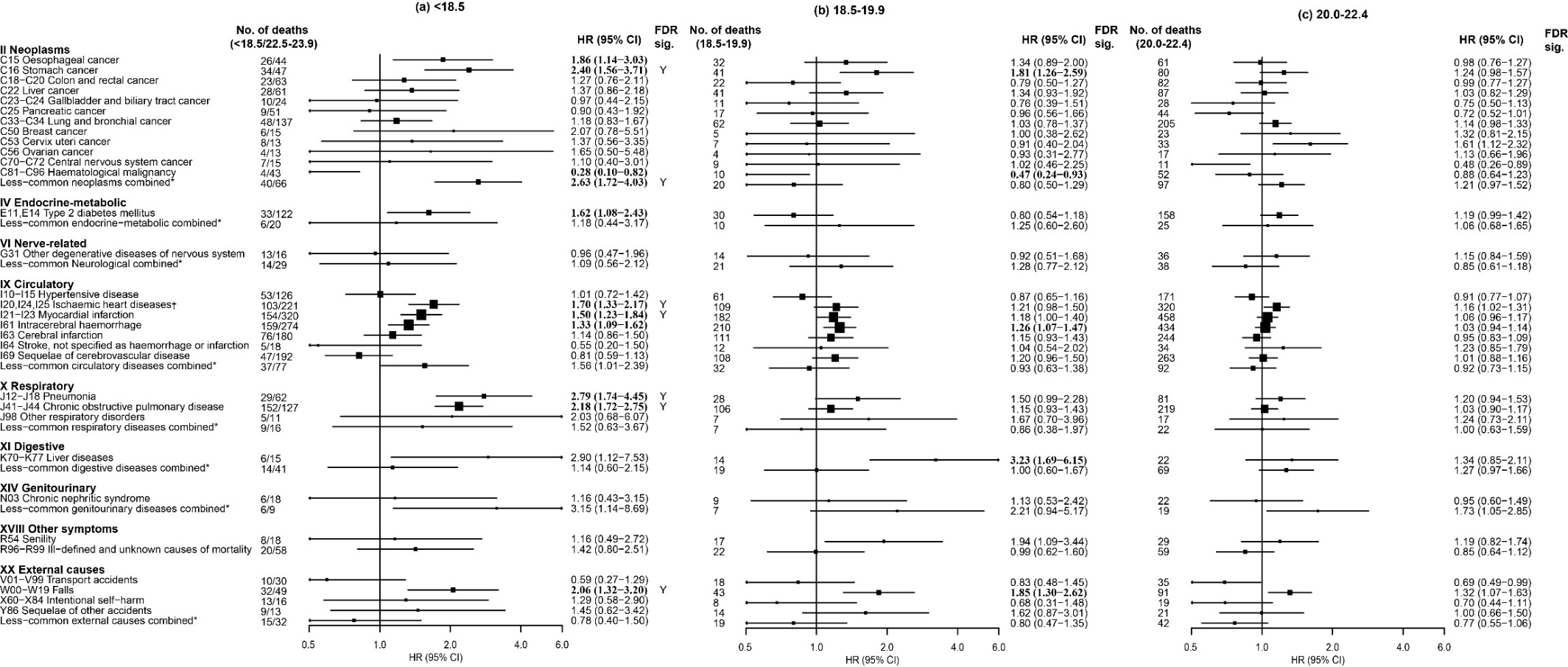


**Figure S2. Adjusted HRs for cause-specific death at or after the age of 70 associated with body mass index**

CI, confidence interval; HR hazard ratio; ICD-10, International Classification of Diseases, 10th Revision.

The group with a BMI of 22.5-23.9 kg/m^2^ was the reference group. The models were adjusted for the same covariates as described in Figure 1. Each solid square represents HR, with the area inversely proportional to the variance of the log HR. The horizontal lines indicate 95% CIs. Bold indicates *P*<0.05. FDR-adjusted *P*<0.05 is denoted by ‘Y’ in the ‘FDR sig.’ column. All *P* values are two-sided.

^*^Included less-common ICD-10 codes within the corresponding ICD-10 chapter that were not individually investigated in the present study.

^†^Ischaemic heart diseases other than myocardial infarction.
